# Ongoing diversification of the global fish pathogen *Piscirickettsia salmonis* through genetic isolation and transposition bursts

**DOI:** 10.1038/s41396-023-01531-9

**Published:** 2023-10-18

**Authors:** Isabel Schober, Boyke Bunk, Gabriela Carril, Heike M. Freese, Nicolás Ojeda, Thomas Riedel, Jan P. Meier-Kolthoff, Markus Göker, Cathrin Spröer, Patricio A. Flores-Herrera, Guillermo Nourdin-Galindo, Fernando Gómez, Constanza Cárdenas, Felipe Vásquez-Ponce, Alvaro Labra, Jaime Figueroa, Jorge Olivares-Pacheco, Ulrich Nübel, Johannes Sikorski, Sergio H. Marshall, Jörg Overmann

**Affiliations:** 1 Leibniz Institute DSMZ-German Collection of Microorganisms and Cell Cultures, Braunschweig, Germany; 2 Laboratorio de Genética e Inmunología Molecular, Instituto de Biología, Pontificia Universidad Católica de Valparaíso, Valparaíso, Chile; 3 German Center for Infection Research (DZIF), Partner Site Braunschweig-Hannover, Braunschweig, Germany; 4 Instituto de Bioquímica y Microbiología, Facultad de Ciencias, Universidad Austral de Chile, Valdivia, Chile; 5 Núcleo Biotecnología Curauma, Pontificia Universidad Católica de Valparaíso, Campus Curauma, Valparaíso, Chile; 6 Laboratorio de Patógenos Acuícolas, Instituto de Biología, Pontificia Universidad Católica de Valparaíso, Valparaíso, Chile; 7 Núcleo Milenio para la Investigación Colaborativa en Resistencia Antimicrobiana (MICROB-R), Santiago, Chile; 8 Institute of Microbiology, Technische Universität Braunschweig, Braunschweig, Germany

**Keywords:** Bacterial genetics, Comparative genomics, Bacterial evolution, Marine microbiology, Bacterial genomics

## Abstract

The management of bacterial pathogens remains a key challenge of aquaculture. The marine gammaproteobacterium *Piscirickettsia salmonis* is the etiological agent of piscirickettsiosis and causes multi-systemic infections in different salmon species, resulting in considerable mortality and substantial commercial losses. Here, we elucidate its global diversity, evolution, and selection during human interventions. Our comprehensive analysis of 73 closed, high quality genome sequences covered strains from major outbreaks and was supplemented by an analysis of all *P. salmonis* 16S rRNA gene sequences and metagenomic reads available in public databases. Genome comparison showed that *Piscirickettsia* comprises at least three distinct, genetically isolated species of which two showed evidence for continuing speciation. However, at least twice the number of species exist in marine fish or seawater. A hallmark of *Piscirickettsia* diversification is the unprecedented amount and diversity of transposases which are particularly active in subgroups undergoing rapid speciation and are key to the acquisition of novel genes and to pseudogenization. Several group-specific genes are involved in surface antigen synthesis and may explain the differences in virulence between strains. However, the frequent failure of antibiotic treatment of piscirickettsiosis outbreaks cannot be explained by horizontal acquisition of resistance genes which so far occurred only very rarely. Besides revealing a dynamic diversification of an important pathogen, our study also provides the data for improving its surveillance, predicting the emergence of novel lineages, and adapting aquaculture management, and thereby contributes towards the sustainability of salmon farming.

## Introduction

**Fig. 1:**
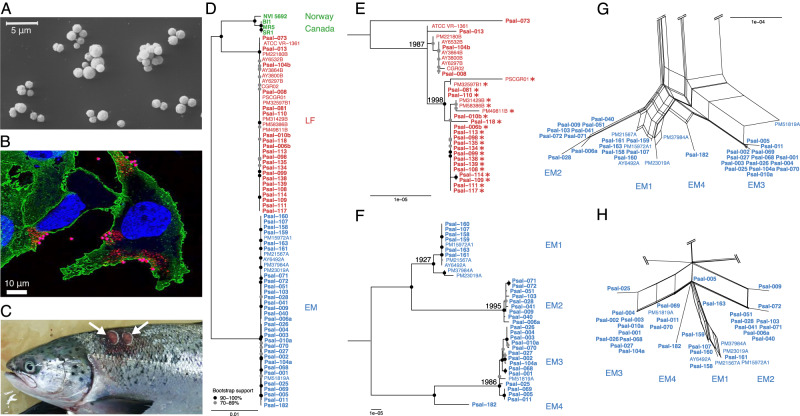
Morphology, pathology and phylogenomics of the intracellular pathogen *P. salmonis*. **A** Scanning electron micrograph of cells of *P. salmonis* ATCC VR-1361 grown in BM3 broth (cell free medium) [73] for 3 days at 18 °C. The image shows the coccoid form of the bacterium and some cell aggregates. **B** Cells of salmon cell line SHK-1 infected with *Piscirickettsia salmonis* strain ATCC VR-1361, 30 min post infection. Bacteria adhere to the cytoplasmic membrane and enter the host cells. Fluorescence signals show localization of antibodies against Clathrin (*green*), *P. salmonis* (*red*), and the nucleus (stained with TO-PRO, *blue*). **C** Two major lesions (white arrows) of the dorsal skin in Atlantic salmon (*Salmo salar*), a typical symptom of piscirickettsiosis. **D** Maximum likelihood phylogenomic tree based on the recombination-corrected core genome of all 73 *P. salmonis* isolates. Chilean isolates of the LF genogroup are depicted in red, those of the EM genogroup in blue, and Norwegian and Canadian isolates in green. Isolates printed in bold were newly sequenced in the course of this study. **E**, **F** Detailed trees of LF and EM genogroups, respectively. Numbers left of nodes give estimates for the begin of divergence (calendar year; only estimates falling within the past 100 years are shown). Dots indicate bootstrap supports of 90-100 (black) or 70-89% (gray). Asterisks denote quinolone-resistant strains with a *gyrA* G259T point mutation. **G**, **H** High resolution subnetworks inferred by SplitsTree NeighborNet analysis from the core genome alignment and from the presence/absence of extrachromosomal element types, respectively, in the genomes of the EM genogroup. For complete phylogenetic networks see Supplementary Fig. S5. Scale bars give substitutions per nucleotide site.

The marine gammaproteobacterium *Piscirickettsia salmonis* was the first Gram-negative intracellular pathogenic bacterium isolated from fish. It was described more than 30 years ago during a severe disease outbreak among farmed coho salmon (*Oncorhynchus kisutch*) in southern Chile where it caused a high mortality of fish stocks [1–3]. From 1991 onwards, the disease was also reported by salmon farms in Norway [4], Scotland and Ireland [5], and Canada [6–8]. *P. salmonis* (Fig. 1A) is the etiological agent of piscirickettsiosis, which continues to be the most prevalent and most lethal bacterial disease of farmed salmon in southern Chile [9], with an overall economic loss reaching US$ 500 million per year [10]. *P. salmonis* causes a systemic infection, particularly in salmonids, by replicating in membrane-bound cytoplasmic vacuoles of infected macrophages and inducing apoptosis [11] (Fig. 1B). Diseased fish exhibit anemia, pale gills, lesions of the skin and liver (Fig. 1C), discolored swollen kidneys and enlarged spleens, necrosis of the intestine, as well as pathological changes in the brain, heart, and ovary [2, 12]. Antibiotic treatments often fail despite the high doses that are used to combat piscirickettsiosis in aquaculture [10, 13], also raising concerns of the horizontal spread of resistance genes.

The genus *Piscirickettsia* and the family *Piscirickettsiaceae*, is still formally assigned to the order *Thiotrichales*, although the latter order is not a monophyletic group [14]. Both, SILVA 16S rRNA and Genome Taxonomy Database (GTDB) taxonomies separate the *Piscirickettsiaceae* into their own order, the *Piscirickettsiales* [15, 16]. In a recent phylogenomic study of the *Gammaproteobacteria*, *Piscirickettsia* genomes form a sister lineage to the *Legionellales* [17].

For the successful prevention and treatment of *P. salmonis* infections, the divergence, evolutionary dynamics, host specificity, and adaptation to interventions of this pathogen must be elucidated [13, 18]. So far, these questions could not be sufficiently addressed since only a very limited number of *P. salmonis* genomes were available [19], only single features were studied in different isolates [6, 20–24], and most strains later were not accessible for follow-up comparisons. The present analysis covers the by far largest data set of high-quality, closed *P. salmonis* genomes generated to date comprising 73 genome sequences, including 55 de novo sequenced genomes of publicly available strains from the new Chilean National *Piscirickettsia salmonis* Strain Collection and originating from Chile, Canada, and Norway.

## Materials and methods

Detailed Materials and Methods can be found in the Supplementary Information (SI).

The complete genome sequences of 51 Chilean, three Canadian and one Norwegian *P. salmonis* isolate sampled from farmed salmonids between 1989 and 2018 were determined in a hybrid approach using long and short read sequencing (SI 1.1–1.3). They were complemented by 18 high quality genomes of Chilean *P. salmonis* isolates and draft genomes of two isolates from New Zealand and one from Hawaii, which were previously published (SI 1.3).

In addition to an initial automatic annotation, coding sequences (CDSs) of special interest were manually analyzed (SI 1.3), and possible virulence factors (SI 1.4), restriction-modification systems (SI 1.4), resistance genes (SI 1.5), and transposases (SI 1.8) were identified by comparison against specific databases. Detection of potential pseudogenes was performed by comparison of consecutive CDSs to identify frameshift fragments, by comparison of lengths of homologous CDSs to identify truncated genes, and by searches for potential gene fragments in intergenic regions (SI 1.4).

Minimum inhibitory concentrations (MICs) for ten different antibiotics were determined for 20 *P. salmonis* isolates, ten from the LF and ten from the EM genogroup (SI 1.5).

The core and pan genomes were calculated based on the homology of the CDSs of the 73 complete genomes (SI 1.4). The phylogenetic breadth of the genus *Piscirickettsia* was assessed using a Maximum Likelihood (ML) phylogenetic tree of 16S rRNA gene sequences from the 73 genomes and all publicly available *Piscirickettsia* 16S rRNA gene sequences (SI 1.6). A genome phylogeny for all complete genomes in the study was reconstructed by ML using a recombination-corrected supermatrix of the concatenated alignments of all single-copy core CDSs (SI 1.6). The phylogeny for the Icm/Dot secretion system was reconstructed from the concatenated *icm*/*dot* gene alignments, also using ML (SI 1.6). To examine species affiliation, digital DNA-DNA Hybridization (dDDH) and Average Nucleotide Identities were calculated (SI 1.6) and the extent of recombination between the genomes was estimated (SI 1.7).

Transposase genes identified in the *Piscirickettsia* genomes were clustered by nucleotide identity, tight clusters with a common ancestor termed transposase types, and origins of transposase types analyzed by comparison against database sequences (SI 1.8). Recent transposase activity was inferred from comparisons of neighboring genes (SI 1.8), chromosome structure and gene order conservation within and between genogroups (SI 1.7).

Extrachromosomal elements (ECEs) were grouped into clusters according to their distance in a tree reconstructed from amino acid sequences. Potential homologous contigs were determined by database searches and the core content, phage content, and the presence of factors required for plasmid mobilization were examined (SI 1.8).

A branch-site model was employed to detect putative positive selection in families of homologous genes. A codon site specific approach was used to identify nucleotide positions in transposase genes exhibiting signs of purifying or diversifying selection during expansion in a single genome (SI 1.9). Gene gains and losses within specific COG categories were modeled using an ML approach. Ancestral character states for the numbers of genes and pseudogenes per homolog group and the number of extrachromosomal elements per genome were reconstructed using maximum parsimony. The correlation between the number of transposase genes and the pseudogene content within genomes was tested with a regression model (SI 1.9).

Tip-permutation tests were performed to test whether the observed character states for geographic origin, year of isolation, and host species of the *Piscirickettsia* isolates were distributed randomly over the topologies of the reconstructed phylogenetic trees. Results are given as *p*-values and retention index *RI* (SI 1.10). In order to determine the spread of *Piscirickettsia* in the oceans, metagenomes in databases were searched for matching sequences (SI 1.10).

## Results and discussion

### Genetic isolation causes divergence of *Piscirickettsia* genomes through multiple and ongoing speciation events

Sizes of the 73 *P. salmonis* genomes (Supplementary Table S1) ranged from 3.25 to 4.15 Mb and chromosome lengths from 3.04 to 3.58 Mb (Supplementary Table S2). Thus, *P. salmonis* has not undergone the extensive genome erosion observed in related intracellular pathogens of the *Legionellales* (1.7–2.83 Mb; SI 2.1). Between 3033 and 4025 coding sequences (CDSs) were predicted for chromosomes. The highest values were observed for Canadian strains (Supplementary Table S2). 1767 CDSs were present in all sequenced *P. salmonis* genomes. This core genome was well covered by our set of strains considering the asymptotic accumulation curve of orthologs (Supplementary Fig. S1). The pan genome was open and comprised 6504 CDSs, mostly due to the four non-Chilean isolates which contributed 1466 additional CDSs.

Previously, two *P. salmonis* clades (LF and EM) had been detected based on sequence comparisons of ribosomal genes [25] and of 19 published genomes [19]. However, additional strains of *P. salmonis* were isolated in Europe and North America [4–6, 8] some of which may represent distinct lineages [5]. Our 73 genomes actually grouped in three distinct and highly supported genogroups (Fig. 1D). The majority of sequences were members of the previously established LF (Fig. 1E) and EM genogroups (Fig. 1F), whereas the four Norwegian and Canadian isolates formed a separate (NC) genogroup that is related to the Chilean LF isolates and in which Canadian strains constitute a distinct subgroup. Most importantly, the present study covered a much larger number of EM isolates than any previous investigation [19, 26], and allowed the detection of four well-separated EM subgroups (EM1 to EM4) with high bootstrap support (Fig. 1F). Three additional, but incomplete genome sequences from New Zealand (*Piscirickettsiaceae* bacterium NZ-RLO1 and NZ-RLO2) and Hawaii (*Piscirickettsia litoralis*´ strain Y2) that had sufficient genome completeness (89–97% of the core genome detected) either grouped with the EM genogroup (NZ-RLO2) or were only distantly related to the established genogroups (Y2, NZ-RLO1) (Supplementary Fig. S2).

Pairwise Average Nucleotide Identity (ANI) and digital DNA-DNA hybridization (dDDH) values for comparisons between the LF, EM, and NC genogroups indicated that they represent up to three separate species. Since the ANI values were right at the threshold of species demarcation, the application of a conservative ANI threshold of 95% (as in GTDB [16]) rather than the established ANI species demarcation range [27] does not reveal this diversification (SI 2.2).

We estimated the timescales of this diversification within *Piscirickettsia*. Due to its rather recent discovery, base substitution rates could not be calculated from the fixed point mutations recorded (SI 1.9). Genomes of the related intracellular pathogen *Legionella pneumophila* accumulate base substitutions at a rate of 2.1 × 10^−7^ site^–1^ year^–1^ (with a 95% credibility interval of 1.7 × 10^−7^ to 2.4 × 10^−7^ site^–1^ year^–1^) [28]. Applying this rate as a proxy for *Piscirickettsia*, the last common ancestors of genogroups EM, LF, and NC date back to 547, 104, and 6554 years B.P., respectively. By comparison, the radiations of subgroups EM1, EM2, and EM3 commenced only 96 to 28 years ago (Fig. 1E, F).

Speciation could have been caused by geographic separation. However, strains from the LF and EM genogroups were isolated from the same region in the same year. Also the detection of the highly related NZ-RLO2 genome sequence in New Zealand revealed that even the EM genogroup is not confined to South America. Furthermore, expanding the analysis to all 238 publicly available *P. salmonis* 16S rRNA gene sequences showed the co-occurrence of different *P. salmonis* lineages even on a global scale (Supplementary Fig. S4). Tip-permutation tests (SI 1.10) showed an only slightly nonrandom distribution of the biogeographic origin of the 16S rRNA gene sequences (*p* = 10^–4^, retention index *RI* = 0.427), most likely caused by the overrepresentation of Chilean isolates rather than by true spatial separation.

Secondly, speciation could be an adaptation to different fish host species, but strains of the different *Piscirickettsia* genogroups have been isolated from the same salmonid species in the same year (e.g., Psal-003/Psal-114, Psal-011/Psal-013, Psal-072/Psal-073, AY6297B/AY6492A; Supplementary Table S1), or even the same host individual (see below). 16S rRNA sequence variants were randomly distributed across fish species (tip-permutation tests yielded *p* = 0.065, *RI* = 0.13), disproving the hypothesis [23] that different phylogenetic clades of *P. salmonis* are adapted to single fish species. For example, EM genotypes infect Atlantic salmon (*Salmo salar*) as well as Pacific salmonids (*O. kisutch, O. mykiss, O. tshawytscha*) (Supplementary Fig. S4).

Despite the lack of geographic separation and host specialization, a phylogenetic network analysis of the core genome showed virtually no network structure between the three genogroups (Supplementary Fig. S5A), indicating that recombination between them is severely restricted, as shown for other sympatric bacterial species [29–31]. At higher resolution, even the four EM subgroups could still be recognized as separate clusters in the SplitsTree network, showing that recombination between them is also limited (Fig. 1G), which is in line with the high bootstrap support of EM subgroups in the phylogenomic tree (Fig. 1F). Indeed, homologous recombination events as detected by ClonalFrameML involved only a low number of small DNA fragments and affected only a minor fraction (<3.5%) of the *Piscirickettsia* genomes (SI 1.7, 2.3). Differences in restriction-modification (R-M) systems have been suggested as one mechanism of genetic isolation and speciation in bacteria [29]. Indeed, we could detect a type IC R-M system and the corresponding, highly methylated, nucleotide sequence motif in Canadian genomes (but not others), as well as a genogroup- and subgroup-specific insertional inactivation of *comEC* and *comF* competence genes in all three genogroups (SI 1.4, 2.3), which represent potential mechanisms of genetic isolation. Based on this cumulative evidence, the three *Piscirickettsia* genogroups represent evolutionarily separated species.

Genogroups remained genetically fully isolated even though they colonize the same host individual: genome sequencing of three supposedly pure original cultures uncovered that each harbored one LF and one EM genogroup strain (Psal-006a/Psal-006b, Psal-010a/Psal-010b, Psal-104a/Psal-104b; Supplementary Table S1). Both members grew in stable laboratory co-culture, without their genomes showing signatures of larger inter-genotype recombination events. Such co-cultures were obtained in three different years from two different salmonid species. Evidently, the three *Piscirickettsia* genogroups constitute distinct and genetically isolated, evolutionary units of which at least two are spatially coexisting. Within the EM genogroup, the speciation process between the four EM subgroups is nascent, while branch lengths, lower sequence identity, and the estimated times of diversification show that the separation of the Norwegian and Canadian strains within the NC genogroup is further advanced.

### Extrachromosomal elements were acquired early, co-evolved with the chromosome, and rarely cause antibiotic resistance

The *P. salmonis* genomes contained a total of 290 extrachromosomal elements (ECEs; Supplementary Table S4A) belonging to 42 different phylogenetic types (Supplementary Fig. S7, SI 2.4). All ECE types were genogroup-specific or even EM subgroup-specific and 286 ECEs were not related to plasmid sequences in other genogroups or any other bacterium, indicating that horizontal transfer among *P. salmonis* genogroups or EM subgroups, or between other bacteria and *P. salmonis* is very rare (Fig. 1H, Supplementary Fig. S5C). Most ECEs had a GC content very similar to the chromosome (39.7-39.8%) and sequences of two ECE types were even detected in the more distant genomes of NZ-RLO1 and Y2. Therefore, ECEs likely were acquired by the ancestors of the genogroups or EM subgroups and subsequently persisted during diversification. Many of the ECEs contained virulence factors and hence may be involved in host adaptation (SI 2.4).

For most *P. salmonis* genomes the CARD Resistance Gene Identifier [32] did not return any perfect or strict hits to known antibiotic resistance genes (Supplementary Table S5). Only ECE type 5, present in three *P. salmonis* strains, carried five known antibiotic resistance markers for tetracycline (*tet*(31)), sulfonamides (*sul2*), chloramphenicol (*cat2*), streptothricin (*sat1*), and aminoglycosides (*aad1*). One strain contained a shorter derivative (ECE type 7) with three of these markers (Supplementary Table S4A). The high sequence similarity of ECE type 5 to plasmids of other fish pathogens [33], its successful conjugative transfer to *E. coli* [33], and the deviating, higher GC contents (53.1 and 55%) point to a recent acquisition of this resistance plasmid through horizontal gene transfer. The four *Piscirickettsia* strains containing the two antibiotic-resistance plasmids did not differ by any SNP in their recombination-corrected core genomes (AY3800B, AY3864B, AY6297B, AY6532B; Fig. 1E) and had been isolated over just three years in the same geographic region in Chile (Supplementary Table S1), suggesting a single acquisition event in this clonal and local subpopulation without spread to other populations. The low prevalence of antibiotic resistance genes in *P. salmonis* is corroborated by a previous study of minimum inhibitory concentrations (MICs) across 292 field isolates from multiple piscirickettsiosis outbreaks which showed a unimodal frequency distribution for oxytetracycline, indicating that resistance against this antibiotic was largely absent [24]. Our laboratory tests of a subset of 20 LF and EM strains confirmed that all strains were susceptible to oxytetracycline, florfenicol, and chloramphenicol (based on established MIC breakpoints; Supplementary Table S6). Collectively, these data contradict the proposal of an effective spread of oxytetracycline resistance among natural fish populations and aquacultures [33] that was invoked to explain the frequent failure of this antibiotic during *Piscirickettsia* outbreaks [7, 10, 33]. Our results were unexpected given the extensive use of oxytetracycline in Chilean salmon farming and the frequent occurrence of *tet* genes in many other gammaproteobacterial genera from fish farms [34]. Likely, the pronounced genetic isolation renders the acquisition rate of resistance plasmids too low to counterbalance plasmid losses, and so far prevented *tet* genes to sweep across *Piscirickettsia*.

**Fig. 2:**
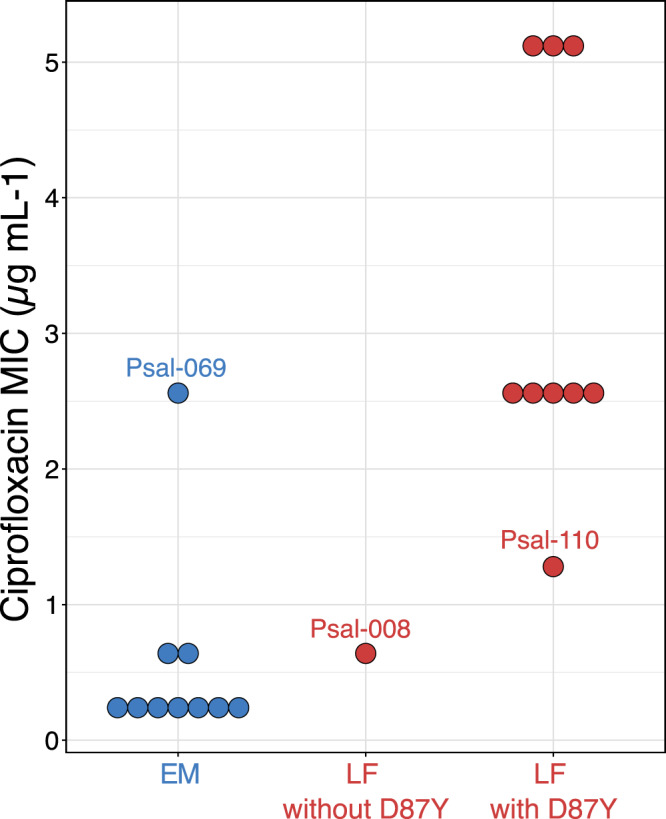
MIC values observed for 10 strains of the EM genogroup (all lacking the D87Y *gyrA* point mutation, Psal-069 displayed a ciprofloxacin-resistant phenotype), and ten strains of the LF genogroup (Psal-008 lacking the *gyrA* point mutation). Psal-069 also did not exhibit any other known resistance mechanisms: mutations in *gyrB*, in *parC*, and *parE* encoding topoisomerase IV, presence of gyrase-protecting Qnr proteins, acetyltransferases, or high expression through mutations in promoter regions of efflux pumps [74].

In contrast to oxytetracycline, resistance to the fluoroquinolone ciprofloxacin was frequently detected (Supplementary Table S6) although it arises only rarely in aquatic environments [35, 36]. The transversion point mutation G259T in the Quinolone Resistance-Determining Region of the *gyrA* gene that causes a D87Y amino acid substitution in the DNA gyrase A subunit and underlies quinolone resistance [37] was observed in 67% of the LF genomes. Strains carrying this mutation also displayed higher MIC values than most other strains (Fig. 2; Supplementary Fig. S8) and formed a single clade in the recombination-corrected core genome phylogeny which we estimated to have emerged around the year 1998 (Fig. 1E, asterisks). By contrast, the *gyrA* point mutation was absent in all our EM strains which also tested sensitive to ciprofloxacin with the exception of strain Psal-069 that seems to feature an unknown mechanism of ciprofloxacin resistance (Supplementary Fig. S8B).

The quinolones oxolinic acid and flumequine had been widely used as first line drugs against piscirickettsiosis in Chilean aquaculture since 1989 [37, 38]. From 2010 on, their use declined and in 2016 amounted to less than 1% of all antibiotics used in aquaculture [39]. Our data are consistent with a scenario in which application of quinolones in salmon farming caused a strong selection of a single LF genotype with a *gyrA* G259T point mutation that subsequently swept through two thirds of the Chilean LF population during a decade of continued usage of these drugs (1998-2010) and even prevailed in the years thereafter. Similar rapid sweeps of *gyrA* point mutations have been documented in human pathogens [40]. *Piscirickettsia* strains also had intrinsic resistances to erythromycin, gentamicin, polymyxin, and beta-lactams (SI 2.4). Contrary to many other pathogenic *Gammaproteobacteria*, antibiotic resistance of *P. salmonis* populations is thus strongly governed by vertical inheritance of chromosome-encoded resistance mechanisms rather than horizontal transfer of resistance genes.

### An unprecedented diversity and expansion of transposases are hallmarks of *Piscirickettsia* evolution

**Fig. 3:**
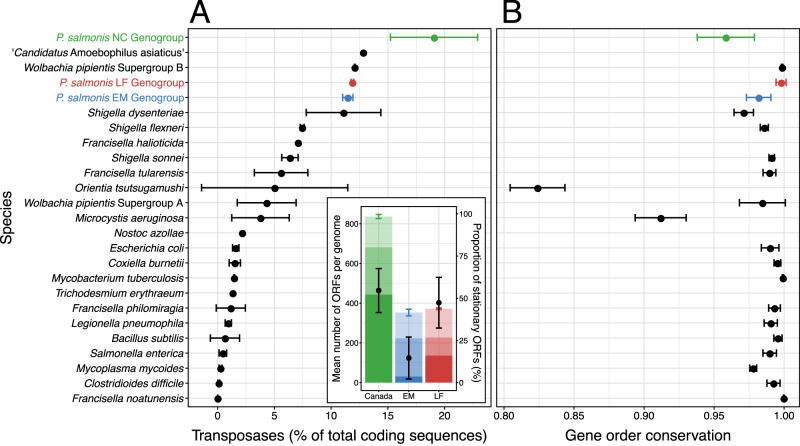
High abundance and genogroup specificity of transposases as well as gene order conservation in *Piscirickettsia* chromosomes. **A** Chromosomal transposase content of *Piscirickettsia* genogroups, close relatives, and of other species with high numbers of transposases. **Insert** depicts the mean number of transposase ORFs which are exclusively present in all members of a single geno- or subgroup (strong shading), present in some of the other genogroups (medium shading), or present in all strains (light shading). Also shown are standard deviations of total numbers of transposases per genome (colored vertical lines) and mean percentages (black dots) plus standard deviations (black lines) of stationary transposases per genome. **B** Gene order conservation (proportion of orthologous coding sequences that are syntenous) for pairwise comparisons of the chromosomes within species/genogroups.

Between 331 and 848 transposase genes were detected in *Piscirickettsia* chromosomes, representing 10.5 – 21.1% of all coding sequences, significantly surpassing the percentages in most other bacterial genomes (<3% [ref. [41]]) including those of related *Gammaproteobacteria* (*Francisella*, *Coxiella*, *Legionella*) (Fig. 3A). The three Canadian isolates had the highest number of transposase genes ever reported for prokaryotes.

The frequency of transposases in prokaryotic genomes increases through horizontal acquisition and by intragenomic replicative (copy-paste) transposition of insertion sequence (IS) elements. *Piscirickettsia* harbors an unprecedented diversity of 106 phylogenetically distinct transposase types (SI 1.8, 2.5). 16 types from four families (IS*1595*, IS*30*, IS*6*, IS*630*; marked in Supplementary Table S8) were closely related (80–100% Amino Acid Identity, AAI) to known transposases from otherwise very distant (genome similarity, 36-37% AAI) bacteria of the phyla *Bacteroidota*, *Actinomycetota*, and *Bacillota* which are dominant constituents of the *S. salar* microbiome [42] and thus likely were exchanged through recent horizontal gene transfer. Over two thirds of the transposase types were present in multiple copies and several transposase types reached copy numbers surpassing those in any other bacterial species [43] (e.g., 248 copies in the Canadian strains; SI 2.5; Supplementary Table S8). This pronounced expansion of transposases is another hallmark of *Piscirickettsia* genomes. Our genome sequencing also provided independent evidence for ongoing transposition events: four of the genomes comprised duplicated regions that occurred extrachromosomally (SI 2.5). The active transposases identified in our work are the most likely cause for the previously observed translocation of a chromosome fragment to a plasmid, transcriptional changes, and the attenuation of cytopathic effects during an extended two-year laboratory cultivation of a *Piscirickettsia* EM strain [44].

**Fig. 4:**
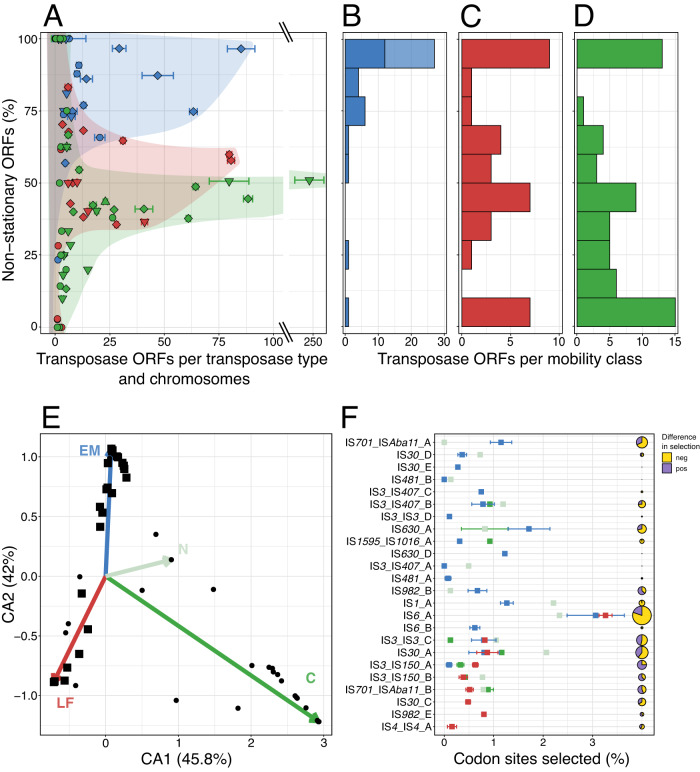
Mobility, expansion, and selection of *Piscirickettsia* LF (red), EM (blue) and NC (green) genogroup transposases. **A** Relation between expansion and mobility. The percentage of non-stationary transposases was calculated as the fraction of genomes per genogroup in which a particular transposase gene ortholog does not have the same neighboring genes. Standard deviations show variations in copy number of single transposase types across genomes. Each symbol represents a transposase type per genogroup under purifying (▼), diversifying (▲), both (◆), or lacking (●), selection pressure. **B**–**D** Numbers of transposases in 10 different mobility classes for the three genogroups. For EM genomes, subgroup-specific or common transposase types are given (light and dark blue shading, respectively). **E** Correspondence Analysis (CA) of the distribution and copy numbers of all 106 transposase types (tips of colored arrows mark the mean geno-/subgroup position in the CA ordination). Black dots mark IS transposase types that were genogroup-specific or did not show evidence of differential selection pressure. Squares mark transposase types under differential selection between *Piscirickettsia* genogroups. **F** Mean percentage of codon sites (per transposase length) of transposase types under differential selection and standard deviations. Pie charts give sum of the Euclidean distances calculated over all positively and negatively selected transposase codon sites (yellow sectors, negative; purple sectors, positive; see SI 1.9) as a measure of differences in selection pressures in different genogroups.

A subsequent analysis of transposase mobility and of signatures of selection pressure on transposases (SI 1.8, 1.9) revealed that transposase activity differs between the *Piscirickettsia* genogroups. To assess transposase mobility, their flanking regions were compared (SI 1.8). This indicated that only 15% of the transposases were stationary in the EM genogroup (insert, Fig. 3A) and high copy number transposases had retained their high mobility even after transposition bursts (Fig. 4A). In particular, all EM subgroup-specific transposases were highly mobile (Fig. 4B, upper light blue bar). In the LF genogroup and Canadian strains, however, about half of the transposases became stationary during radiation (insert, Fig. 3A) and high mobility was observed only for low copy number transposases whereas highly expanded transposases had limited mobility (Fig. 4A, C, D). This corresponds to the higher fraction of truncated transposases found in LF and NC genomes (Supplementary Table S9A). Transposition can also be attenuated by the formation of stable hairpin structures by transcripts repressing their translation. Five of the low copy number transposases contained such hairpins (see SI 2.5). Genogroups not only differed in their specific transposase inventories (dots in Fig. 4E), but also in the extent and direction of selection pressure of the 24 transposase types that occurred across genogroups (squares in Fig. 4E, F). Particularly transposases with strong expansion harbored sites that were under purifying and/or diversifying selection pressure (indicated by triangles or diamonds in Fig. 4A).

During genome evolution, copies of the same IS element can serve as recombination sites for large-scale genome rearrangements. Transposition also mediates the acquisition of novel genes, pseudogenization, or changes in the regulation of preexisting genes. Within the *Piscirickettsia* LF and EM genogroups, gene synteny was largely maintained and gene order conservation (GOC) values were similar to closely related bacterial species with a much lower transposase content (Supplementary Fig. S11A, B). *Piscirickettsia* chromosomes also lacked the highly broken GC skew patterns that are typical for species with highly active transposases like *Orientia tsutsugamushi* and *Microcystis aeruginosa* [45, 46] (Fig. 3B, Supplementary Fig. S12). However, genome synteny was unusually decreased within the NC genogroup and also between the three *Piscirickettsia* genogroups considering their genomic relatedness (Supplementary Fig. S11B, C). Evidently, major genome rearrangements occurred during the differentiation into three *Piscirickettsia* genogroups as well as during the split between Norwegian and Canadian genomes in the NC genogroup, but must have considerably slowed down during subsequent radiation of the LF and EM genogroups.

Up to 20% of the CDSs in *Piscirickettsia* genomes represented pseudogenes. While most prokaryotic genomes contain only few pseudogenes (1–5% of all CDSs [47]), high percentages of pseudogenes of up to 50% or more have been observed in genomes of facultative intracellular pathogens or recent symbionts [48–51] and were caused by an extreme multiplication of IS elements. Genomes of the EM genogroup had the lowest and Canadian genomes the highest numbers and percentages of pseudogenes. The dominant mechanism of pseudogenization was truncation. Transposases were found to flank pseudogenes and shorter pseudogene fragments in intergenic regions more frequently than would be expected if they were distributed randomly across the genome (Supplementary Table S9A, B; SI 2.5).

Therefore, the role of transposases in the evolution of *Piscirickettsia* was assessed by a phylogenetic regression analysis of the effects of transposases on overall gene and pseudogene content using BayesTraits (SI 1.9). For the EM genogroup, the numbers of functional transposases had the highest explanatory power for the variance in numbers of non-transposase genes (*R*^2^ = 0.64), suggesting that transposases were most relevant for the acquisition of novel genes. By contrast, the numbers of functional transposases in the LF genogroup had the highest explanatory power for the variance in numbers of non-transposase pseudogenes (*R*^2^ = 0.50), and hence mostly affected pseudogenization. IS elements therefore represent major drivers of genome evolution in *Piscirickettsia* and contributed towards the differentiation of the genogroups.

### Genogroup-specific evolution of gene content and functional implications

**Fig. 5:**
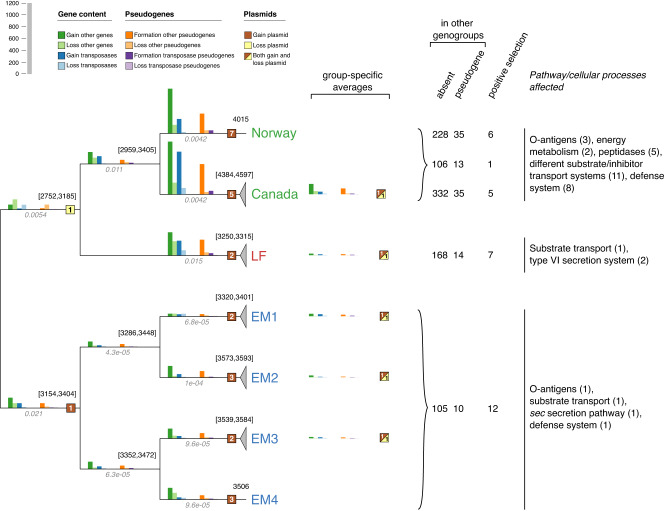
Changes in gene content during the evolution of *Piscirickettsia* geno- and subgroups. Cladogram with a quantitative comparison of changes in gene content (bar charts with blue and green colors for transposases and other genes, respectively), formation and loss of transposases and other pseudogenes (bar charts with purple and orange colors), and of changes in extrachromosomal elements (ECEs; squares shaded with brown and yellow color) during the different phases (i.e. subbranches) of the speciation of the genus Piscirickettsia. For terminal radiations in the individual genogroups or EM subgroups, average values for gains and losses are provided next to geno-/subgroup designations. Missing squares indicate that no changes occurred on the respective branches. Numbers to the upper left of each node give the lower and upper values of the reconstruction of the ancestral genome sizes or actual genome sizes where terminal nodes are displayed. For comparative reasons, branch lengths in substitutions per site are printed in italics below the branches. Numbers of genes that are group-specific due to their absence or pseudogenization in other genomes or showing signs of positive selection are given for the three genogroups and, separately, for the Norwegian and Canadian strains. Also, major pathways and cellular processes inferred from the annotated genes as affected by the absence, pseudogenization or positive selection are provided for the three genogroups (see text for further details). Another version of the figure utilizing a phylogeny with branch lengths can be found in Supplementary Fig. S15.

The distribution of homolog groups across all *Piscirickettsia* genomes revealed considerable geno- and subgroup-specific gene inventories (SI 1.9, 2.6). Therefore, changes in gene content, pseudogenization, extrachromosomal elements, and genogroup-specific positive gene selection during *Piscirickettsia* evolution were quantified and the resulting group-specific gene functions were determined (Fig. 5). An initial quantification of gene gains and losses by BadiRate showed unusually large changes in the COG L gene families (encompassing transposases). Therefore, gene gains and losses were quantified separately for transposase and non-transposase genes.

Gene gains exceeded losses during the evolution of genogroups and EM subgroups all the way up to their most recent terminal radiations (Fig. 5). At the same time, the high proportion of pseudogenes indicates ongoing gene losses, so the overall dynamics of *Piscirickettsia* genomes is truly remarkable. The net gain of genes is in line with *Piscirickettsia* genome sizes (3.25–4.15 Mb) that clearly exceed that of the last common ancestor shared with the *Legionellales* (2 Mb [17]). Whereas in the LF genogroup most genes were gained before the divergence of its members, major gene gains in the EM genogroup were assigned to the six short branches leading to the four EM subgroups and hence were associated with subgroup diversification (Fig. 5). The largest gains of specific genes were detected for the genomes of the Norwegian and Canadian isolates. Plasmids were gained mostly during early evolution of *Piscirickettsia* whereas plasmid gains and losses became balanced during the recent divergence of the Canadian strains, the LF genogroup, and strains of the EM subgroups. The resulting specific gene inventories ranged from 115 genes in the EM genogroup up to 367 genes in the Canadian genomes (Fig. 5; SI 1.9). Between 8 and 13% of these genes were still detectable as pseudogenes in the genomes of at least one other genogroup. In addition, up to 12 core genes were found to contain genogroup-specific polymorphisms when employing the aBSREL tool [52], indicating a positive selection (Fig. 5).

Some *Piscirickettsia* strains have been shown to differ in their levels of virulence and resistance to the salmon complement system [20, 23]. In the fish pathogen *Vibrio anguillarum*, O-antigen polysaccharides have been shown to mask surface-located molecular patterns, preventing phagocytosis by skin epithelial cells of fish, and also provide resistance to antimicrobial factors [53]. In *Piscirickettsia*, the UDP-glucose-4-epimerase gene for O-antigen galactofuranose synthesis was positively selected in the EM genogroup (homolog family group 92; Supplementary Table S10). Only Canadian strains contained two genes involved in L-fucose synthesis (groups 2960, 2961), or in 3,6-dideoxyhexose synthesis (CDP-4-dehydro-6-deoxyglucose reductase; group 2962). In O-antigen polysaccharides, fucose can be bound by C-type mannose-specific host lectins [53]. The 3,6-dideoxyhexoses ascarylose, abequose, and tyvelose are other major antigen determinants which so far were only known from a limited number of enterobacteria (*Salmonella* and *Yersinia*) [54]. Vice versa, Canadian strains lacked the biosynthetic pathway for pseudaminic acid which has experimentally been shown to occur in *Piscirickettsia* lipopolysaccharides [55]. Many of the other genogroup-specific or specifically selected genes that were functionally annotated are also associated with host-pathogen surface interactions (O-antigen synthesis, OM proteins, peptidases, defense systems, transporters; Fig. 5, SI 2.6). Our findings imply distinct surface antigenic structures of individual *Piscirickettsia* geno- and subgroups.

Another virulence factor with group-specific distribution in *Piscirickettsia* is the Icm/Dot Type IVB secretion system [11, 56]. This secretion system constitutes the main virulence factor in the *Legionellales*, including the intracellular parasites of ruminants, arthropods, ticks, and amoebae (*Aquicella*, *Rickettsiella/Diplorickettsia*, *Candidatus* Berkiella*´*). In *Piscirickettsia*, its expression is increased after the uptake by macrophages and upon acidification [3, 11, 57] while mutants are attenuated in the fish host [22]. Aside from the secretion system itself, we also detected 10 different genes encoding *icm/dot* effectors (Supplementary Table S11A).

The unique feature of all closed *P. salmonis* genomes is the duplication or triplication of the *icm/dot* gene cluster (Supplementary Fig. S16). Higher numbers of *icm/dot* gene clusters in the LF strains and the Norwegian isolate in combination with a higher degree of mutational inactivation by frameshifts in the LF genogroup (SI 2.6) may at least partly explain the differences in virulence observed for different *Piscirickettsia* isolates [20, 21, 23]. The *icm/dot* gene clusters of *P. salmonis* had strong structural similarities to that of *L. pneumophila* and *Coxiella burnetii* and likely originated from a common ancestor (Supplementary Fig. S16A, B). Our phylogenetic reconstruction revealed two early duplication events prior to the divergence of the *Piscirickettsia* genogroups, yielding three gene clusters, here denoted A, B, and C (Supplementary Fig. S16B). *Piscirickettsia* strains Y2 and NZ-RLO1, which are less closely related to the other strains as the latter are to each other, contained the three *icm/dot* gene clusters. Lineage Y2 may have diverged from the other genomes about 200 million years ago [17], which is later than the occurrence of the last common ancestor of all bony fishes (Osteichthyes; 420 million years ago [58]). The acquisition of multiple *icm/dot* gene clusters therefore could have occurred during the colonization of bony fishes by *Piscirickettsia*. The presence of cluster C in strain NZ-RLO2 together with its absence in the very closely related 36 EM genomes and the three Canadian genomes (Supplementary Fig. S2) indicates two recent, independent losses of the ancient *icm/dot* cluster C just prior to the radiation of the EM genogroup and of the Canadian subgroup, respectively. Our observation of *icm/dot* gene clusters in two extrachromosomal circular translocatable units of the EM strain Psal-072 and LF strain Psal-073 (see above, Supplementary Fig. S10) indicate two ongoing, independent translocation events. Likewise, a mobilization of the *icm/dot* gene cluster on composite transposons may have caused the early duplication of these clusters in *Piscirickettsia*.

### Unexpected diversity, habitat range, and worldwide distribution of *Piscirickettsia*

We finally assessed the geographic distribution of *Piscirickettsia* beyond the available isolates, and determined to which extent cultivated strains are representative for the diversity within the genus.

**Fig. 6:**
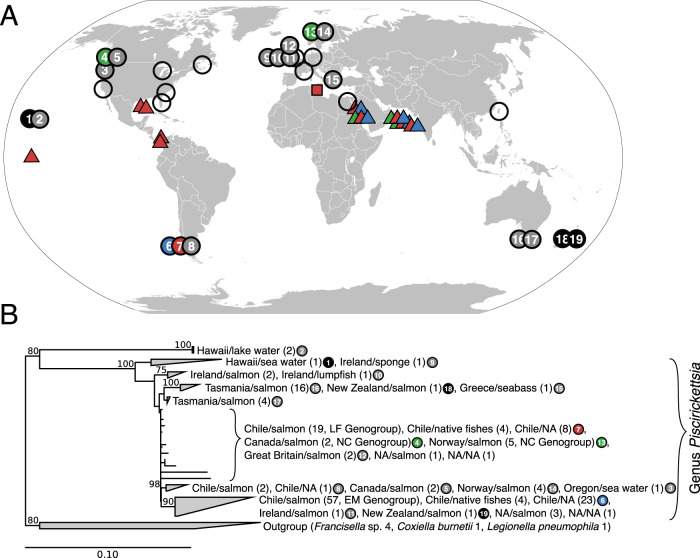
Global biogeography and phylogeny of the genus *Piscirickettsia*. **A** Geographic distribution of *P. salmonis*, *Piscirickettsia* sp., and similar organisms detected by 16S rRNA gene sequences (*filled symbols*; compare Supplementary Fig. S4) or by histopathology, immunological testing, or 16S-ITS-23S rRNA sequencing without available 16S sequence information (*open symbols*; compare Supplementary Table S12). Colored circles indicate origins of the isolates studied in the present work, including 33 Chilean strains of the LF genogroup (red), 36 Chilean strains of EM genogroup (blue) and one Norwegian and three Canadian strains (NC genogroup; green). Black filled circles show origin of isolates with short read draft genomes, gray filled circles the origin of 16S rRNA gene sequence database entries. Triangles indicate locations at which *Piscirickettsia* sequences were detected in marine metagenomes and the rectangle the detection of *Piscirickettsia* sequences in contaminated draft genomes from bacterial isolates from the rhizosphere of *Limoniastrum monopetalum*. **B** Maximum likelihood phylogenetic tree inferred from all available database 16S rRNA sequences deposited in GenBank as *P. salmonis*, *Piscirickettsia* sp. or *Rickettsia*-like´ organisms. Coherent groups of sequences were collapsed into clades. For each clade or genogroup, the number of sequences originating from specific countries, hosts or environments is given in parentheses. NA indicates lack of information on origin. Numbers at nodes give bootstrap values in %; only values greater than 70% are shown. The fully resolved tree is shown in Supplementary Fig. S4.

The set of 238 different 16S rRNA sequences of *P. salmonis* and related bacteria showed a broad geographic distribution (Fig. 6A, circles) and were detected mostly in farmed fish belonging to four salmonid species, but also in 14 different non-salmonid species as well as one marine sponge (Supplementary Fig. S4, Supplementary Table S12). Nearly full-length sequences (>1300 bp; *n* = 211) were used to determine the phylogenetic divergence. Aside from two phylotypes that originated from Hawaiian lake water (LA1-B44N and LA7-B48N; Supplementary Fig. S4), all sequence types had pairwise similarity values >94% and hence can be considered members of the genus *Piscirickettsia* based on the accepted similarity threshold for bacterial genera [59]. Within the genus, a considerable variety of lineages and five distinct phylogenetic clades with stable bootstrap support can be recognized (Fig. 6B). Considering that the LF and NC genogroups cannot even be distinguished with regard to their 16S rRNA gene sequences and taking into account the open *Piscirickettsia* pan genome, the number of species must at least be twice the number deduced from our genome comparison of the 73 available closed genomes.

*P. salmonis* was so far only reported sporadically and mostly for marine waters adjacent to salmon farms [60]. However, BLAST searches using all protein-coding genes of four representative genomes of *Piscirickettsia* LF, N, C, and EM groups against the EBI metagenome database MGnify returned hits for 76 different groups of homologs of *Piscirickettsia* hypothetical proteins. Also hits for nine transposase types were found, reported from 10 different locations in five distant oceanic provinces (triangles in Fig. 6A, Supplementary Table S12; all hits were in the TARA Ocean dataset). *P. salmonis* thus is much more wide-spread in the marine environment than previously thought. A global dispersal of different *P. salmonis* genogroups is in line with (i) over 150 years of successful worldwide introduction of salmonids and of extensive shipments of salmonid eggs [61], (ii) the continuous release of *P. salmonis* from infected fish via bile, feces, and urine into seawater [62] combined with the long-term survival of free-living cells for up to 40 days in situ [63], (iii) the rapid spread of piscirickettsiosis through surrounding waters between fish from different stocks, hatcheries, and freshwater rearing facilities [64], and (iv) the horizontal transfer of *P. salmonis* between fish individuals that was shown experimentally [2, 20].

An entirely unexpected finding was the detection of numerous contigs which were identical to chromosomal regions of *Piscirickettsia* in two draft genomes of *Bacillus halotolerans*, indicating a contamination of the sequenced *Bacillus* culture by *Piscirickettsia* cells that must have been present in the inoculum that originated from the rhizosphere of *Limoniastrum monopetalum* (Grand Statice), a native plant of Mediterranean saltmarshes and sandy beaches often in direct contact with seawater during high tides (Fig. 6A, red square). The contigs perfectly matched the genome of *P. salmonis* Psal-002 of the EM genogroup, comprised a large variety of different functional genes, and covered a considerable portion (35,945 and 307,389 base pairs, respectively) of the two deposited, contaminated *Bacillus* genomes. While this initial finding needs to be substantiated by systematic screenings in the future, it supports the notion of a very broad environmental distribution of *Piscirickettsia* and indicates that this fish pathogen also persists in a viable state in previously unrecognized habitats.

### Implications for the future management of *Piscirickettsia*

Salmonid farming currently accounts for 90% of all industry-related antibiotic use in Chile [6], applying florfenicol and oxytetracycline for the prophylaxis of piscirickettsiosis [37, 39]. Despite the high dosage, antibiotics treatments often failed over the past 30 years [7, 10, 13, 33], which has been attributed to resistance formation [33, 64], but based on our results must be caused by other factors. As shown in our study, antibiotic resistance in *Piscirickettsia* is mostly intrinsic, clearly absent for florfenicol and oxytetracycline, and typically does not involve the horizontal acquisition of antibiotic resistance seen in other *Gammaproteobacteria*, that also causes concerns for human health. Instead, a continuous reinfection of farmed salmon by *Piscirickettsia* originating from the previously unrecognized reservoirs in coastal environments, potentially even the nutrient-rich rhizosphere [65] of marine coastal plants, may account for the repeated failure of antibiotic treatments. Once acquired, however, antibiotic resistance could sweep efficiently through *Piscirickettsia* populations as shown by our data on quinolone resistance in the LF genogroup. In light of the unpredictable outcome of antibiotic treatments, the worldwide increase in salmon production, and the widespread distribution of *Piscirickettsia*, alternative strategies were recently called for [13] and are clearly needed for the future management of piscirickettsiosis. Such strategies could encompass dedicated preventive measures including surveillance, predictions of outbreaks, and improved aquaculture design.

The pronounced genomic diversification in the genus *Piscirickettsia* determined in the present study was previously unknown due to the very limited number of closed genome sequences and the strong underrepresentation of EM strains. The additional groups of *Piscirickettsia* that were uncovered by our analysis of available 16S rRNA and metagenome sequences still await future isolation and characterization. The genomic information gathered in the current work provides the means to develop methods for the specific monitoring of these different groups. The genogroup and subgroup-specific stationary transposases offer particularly promising targets for high throughput PCR-based fingerprinting to circumvent the tedious isolation and typing of individual strains [66] and, together with other genogroup-specific genes, would allow the quantification of bacterial loads by qPCR. Furthermore, our analysis of the genogroup-specific gene contents revealed consistent differences in surface antigen synthesis pathways and other virulence-associated traits. This type of information can potentially serve to predict the severity of future piscirickettsiosis outbreaks in salmon aquaculture.

We detected *Piscirickettsia* genomic fragments in datasets from diverse coastal and open ocean environments. To date, the major environmental reservoirs of this important fish pathogen could not be identified. In this respect, sensitive detection methods targeting group-specific genes would also enable a systematic screening of environmental samples and could help to design and operate salmon aquacultures in suitable locations and thus improve their sustainability.

*Piscirickettsia* is in an intermediate stage of host adaptation and lacks the hallmarks of an obligate relationship such as a reduced genome size and absence of IS elements [67, 68]. Bacteria with high transposase content typically are intracellular pathogens or symbionts; a content >10% has only been reported for *Shigella dysenteriae*, the amoebae symbiont *Candidatus* Amoebophilus asiaticus´ (*Bacteroidota*), the human pathogenic alphaproteobacterium *Orientia tsutsugamushi*, and the mollusc pathogen *Francisella halioticida* [45, 69, 70]. The unusually high transposase content of *Piscirickettsia* indicates an earlier stage of host adaptation and likely is caused not only by genetic isolation, but also by small population sizes and population bottlenecks that render the loss of genes without selective advantage less efficient [71, 72]. Speciation is still ongoing within the NC genogroup and the EM genogroup has commenced to split into four different subgroups just over the past centuries. Transposition has been one of the drivers of early evolution of *Piscirickettsia* and could be shown to be ongoing particularly in the EM genogroup. Like the EM2 and EM3 subgroups that have emerged over the past decades and evolve rapidly, novel *Piscirickettsia* pathogens may be expected in the near future. This is of specific relevance to piscirickettsiosis outbreaks in Chilean aquaculture because of the higher virulence of EM genogroup strains which cause acute and severe systemic and hemorrhagic disease and a higher cumulative mortality [20, 21].

### Supplementary information

Supplementary Information

Supplementary Figures

Supplementary Tables

## Data Availability

The genomes sequenced during the current study are available on NCBI GenBank. Nucleotide accession numbers of all newly sequenced genomes and *Piscirickettsia* genomes downloaded from GenBank for these analyses are listed in Supplementary Table S1. Nucleotide accession numbers of reference genomes of other species used for transposase and gene order conservation analyses are listed in Supplementary Table S13. 16S rRNA gene nucleotide accession numbers of *Piscirickettsia salmonis, Piscirickettsia sp., or Rickettsia-like organisms* used for the phylogeny of the genus *Piscirickettsia* are listed in Supplementary Table S14.
