# Supplementary material for: Ongoing diversification of the global fish pathogen Piscirickettsia salmonis through genetic isolation and transposition bursts
Source: ISME J. 2023 Oct 18;17(12):2247–58. doi: 10.1038/s41396-023-01531-9 (PMC10689435; doi:10.1038/s41396-023-01531-9)
Supplement: Supplementary file 1 — Supplementary Information [file 41396_2023_1531_MOESM1_ESM.docx]

**SUPPLEMENTARY INFORMATION**

1. **Supplementary Materials and Methods**

**1.1 Origin and cultivation of *Piscirickettsia salmonis* isolates**

The complete genome sequences of 55 *P. salmonis* isolates were determined during the course of this study. 51 Chilean isolates had been deposited in the Chilean National Strain Collection of *Piscirickettsia salmonis* (SERNAPESCA, 2832 Victoria Street, Valparaíso; and Pontificia Universidad Católica de Valparaíso, Avenida Universidad 330, Curauma, Valparaíso, Chile, as well as Laboratory for Molecular Fish Biology, Institute of Biochemistry and Microbiology, Universidad Austral, Isla Teja Campus, Valdivia, Chile). Three Canadian isolates were provided by Dr. Simon Jones (Pacific Biological Station of Fisheries and Oceans Canada, Nanaimo, B.C., Canada), and one Norwegian isolate was contributed by Dr. Duncan Colquhoun (Norwegian University of Life Science, Ås, Norway). All isolates originated from farmed salmonids between 1989 and 2018 and represented different major outbreaks. Other non-Chilean isolates reported in literature could not be acquired as they had either been lost, were overgrown by contaminants or could not be regrown from stocks.

Isolates were cultured from cryostocks stored at -80 °C and incubated on IFOP-PsM11 [1] or tryptone soy agar plates with hemoglobin (Austral-TSHem [2]) at 18 °C for up to 10 days. After incubation, colonies were transferred to 6 mL of Austral-SRS broth [3] and grown at 18°C and 100 rpm until cultures reached an OD_600_ of 1.

**1.2 Quality control and identification of strains**

All cultures and DNA extracts were tested to confirm their identity and to check for contaminations. Gram-staining was performed according to standard procedures [4] and examined using a Leica DM5000B Microscope (100x objective, immersion oil). The Salmonid Rickettsial Septicemia (SRS) Immunofluorescent test (Ango Diagnostic, Santiago de Chile) was employed to specifically stain *Piscirickettsia*. 20 µL of each bacterial culture was dried on a glass microscopic slide and fixed with cold acetone for 10 min at room temperature, followed by two washes with sterile PBS (NaCl, 1.369 M; KCl, 0.027 M; Na_2_HPO_4_, 0.101 M; KH_2_PO_4_, 0.018 M). 100 µL of Oligoclonal reagent (Ango Diagnostic; diluted 1:100) were added and samples incubated in a moist chamber at room temperature for 30 min. Samples were washed twice for 5 min with washing solution (diluted 1:25 with distilled water), before 100 µL of anti-IgG-FITC solution (diluted 1:100) were added and samples were incubated for 30 min in the dark at room temperature in a moist chamber. Subsequently, samples were again washed twice for 5 min with washing solution. A drop of DABCO (1,4-diazabicyclo[2.2.2]octane) fluorescence mounting medium (Agilent Technologies, California, United States) was added, samples were dried and covered with a sterile cover slip, and viewed in a Nikon Eclipse Ti Fluorescence Microscope using a 60x objective.

Two different PCR assays were performed to confirm the identity of *P. salmonis* cultures. Firstly, a species-specific PCR targeting the internal described spacer was performed, using the primers RTS-1 (223F) (5’-TGATTTTATTGTTTAGTGAGAATGA-3’) and RTS-4 (5’-ATGCACTTATTCACTTGATCATA-3’) [5]. Secondly, a nested PCR was performed which comprised an initial amplification of the 16S rRNA gene with bacterial primers 27F (5’-AGAGTTTGATCCTGGCTCAG-3’) and 1487R (5’-ACGGATACCTTGTTAGCAGTT-3’), followed by a second amplification with the species-specific primers PS2S(223F) (5′-CTAGGAGATGAGCCCGCGTTG-3′) and PS2AS(690R) (5′-GCTACACCTGCGAAACCACTT-3′) [6].

**1.3 DNA extraction, genome sequencing, assembly, annotation**

*P. salmonis* cultures were grown in Austral-SRS broth, centrifuged at 6,000 xg for 15 min, and DNA was extracted with the QIAGEN Genomic-tip 100/G kit (Qiagen, California, U.S.) following the instructions of the manufacturer. The concentration and quality of genomic DNA were determined with a NanoDrop ND-1000 spectrophotometer, and fragment length evaluated in agarose gels (0.8% w/v).

Genomes were sequenced on the PacBio *RSII* (Pacific Biosciences, Menlo Park, CA). Genome assembly was carried out using the ‘RS_HGAP_Assembly.3’ protocol in the SMRT Portal software suite version 2.3.0. The resulting contigs were trimmed and circularized by cutting off overlapping ends. Chromosomes were adjusted to *dnaA* as the start point, and extrachromosomal elements according to the predicted replication or partitioning proteins. The SMRT Portal ‘RS_BridgeMapper.1’ protocol was used for long read correction. All genomes were also sequenced on an Illumina MiSeq or NextSeq and the obtained reads were used to correct the typical insertion and deletion errors from PacBio sequencing, using the Burrows-Wheeler Aligner version 0.7.12-r1039 [7] and VarScan v2.3.6 [8]. This yielded high quality complete genome sequences with a predicted error rate per genome of <10^-6^ (QV60). For gene calling and annotation the Prokka pipeline version 1.8 [9] was employed. The genomes are deposited in NCBI GenBank and the accession numbers are provided in Suppl. Table S1.

Of the 20 closed genomes of *P. salmonis* available in NCBI GenBank [10] in January 2020 18 high quality genome sequences were included in our analysis. Genome EM-90 (CP033937.1 - CP033945.1) was not included as it is a duplicate to Psal-002 and extrachromosomal elements are incorrectly assembled. Genome PM25344B (CP013821.1 - CP013825.1) was dismissed due to quality concerns. In comparative analyses not requiring complete genomes we also included three draft genomes that were available and sufficiently complete (89-97% of the core genome were detected). These latter sequences originated from New Zealand (*Piscirickettsiaceae* bacterium NZ-RLO1, NZ-RLO2) and Hawaii (`*Piscirickettsia litoralis*´ strain Y2). Metadata of all strains and genome sequences are listed in Suppl. Tables S1 and S12. In order to ensure comparability of the previously published 18 genome sequences downloaded from GenBank, the start points of their chromosomes and extrachromosomal elements were readjusted and the genomes were reannotated using Prokka as described above.

For gene identification, the amino acid sequences of genogroup-specific and strain-specific coding sequences were compared against the UniProtKB/Swiss-Prot database [11] using BLAST [12] and additionally annotated by KEGG orthology using BlastKOALA [13]. All cellular pathways in which ≥ 3 of the involved genes were annotated and exhibited genogroup-specific differences in the presence/absence, pseudogenization or selection were analyzed in more detail.

**1.4 Core and pan genome analysis, identification of virulence factors, R-M systems, and pseudogenes**

The core and pan genomes of the set of 73 closed genomes were calculated for all coding sequences of the complete genomes, including extrachromosomal elements, using Proteinortho version 5.16b [ref. 14] with the --singles option and default values for all other parameters. The core genome was obtained as the number of homolog groups with at least one member in each genome. The pan genome was counted as the number of all homolog groups detected. Pan and core genome accumulation curves were drawn using the Proteinortho_curves v1.0 script with 1,000 iterations (available on GitHub at <https://github.com/isabelschober/proteinortho_curves>). Genes unique to specific groups of strains were determined using custom Python scripts.

To identify possible virulence factors in the *Piscirickettsia* genomes, coding sequences from each genome were compared against the ‘VFDB_setB_pro.fas’ data set downloaded from the Virulence Factor Database (VFDB, accessed February 2018) [15, 16] using best BLAST hits [12] with maximum e-values of 1E-30. The resulting hits were additionally filtered applying 30% amino acid identity and 60% query coverage as minimum criteria. In order to resolve the complete Icm/Dot Type IVB secretion system, open reading frames (ORFs) in the vicinity of genes identified by VFDB were blasted manually against the whole non-redundant (‘nr’) protein sequence database of the NCBI and screened using NCBI CD-Search with default parameters. For comparison, Icm/Dot component protein sequences of *Legionella pneumophila* subsp. *pneumophila* strain Philadelphia 1 (GenBank AE017354.1) and *Coxiella burnetii* RSA 493 (GenBank AE016828.3) were used.

Restriction-modification (R-M) systems were identified based on KEGG annotation and by Protein BLAST against the REBASE restriction enzyme database (rebase.neb.com) [17] applying strict thresholds (e-value ≤10^-30^, identity ≥30%, query coverage≥60%). For all newly sequenced genomes, the methylome was determined based on SMRT sequencing interpulse duration in the SMRTPortal 2.3.0 using the ‘RS_Modification_and_Motif_Analysis.1’ protocol with default parameters and a minimum modification QV of 30.

Three complementary methods were employed to identify pseudogenes that arose by different mechanisms in the *Piscirickettsia* genomes. Firstly, frameshift mutations that resulted in two or more consecutive pseudogene fragments were determined employing NCBI’s ‘Microbial Genome Submission Check’ (https://www.ncbi.nlm.nih.gov/genomes/frameshifts/frameshifts.cgi), which identifies pseudogenes as two neighboring ORFs producing BLAST hits against the same subject each covering ≤90% of it. Secondly, pseudogenes that arose through truncation of genes by IS insertion, terminal frameshift mutations, or premature stop codons followed by deletions, were identified as proteins that had a shorter length (<80%) compared to their orthologs [18]. For this, BLASTp searches were performed against the protein sequences of the three *Piscirickettsia* genogroups, but also against the proteins encoded by the genomes of close relatives (four genomes from each of the following species: *Legionella pneumophila*, *Coxiella burnetii,* *Francisella noatunensis*, *F. philomiragia*, *F. tularensis*; as listed in Suppl. Table S13). Hits with an e-value ≤1E-30 and identity ≥30% over a length of ≥90% of the query sequence were counted as homologs. Proteins shorter than 80% of the length of the shortest homolog in at least one genus other than *Piscirickettsia*, or shorter than 80% of the mean lengths of homologs in at least one of the *Piscirickettsia* genogroups, were identified as truncated. Thirdly, non-coding, short remaining gene fragments were identified in intergenic regions by searching for BLASTx hits against all coding sequences in *Piscirickettsia* genomes or in the genomes of the related species using e-values ≤1E-30 and a minimum length of 100 bp.

**1.5 Antibiotic resistance genes and minimum inhibitory concentrations**

Minimum Inhibitory Concentrations (MICs) for ten different antibiotics used in veterinary care were determined by broth microdilution antimicrobial susceptibility assays [1]. Ten isolates from the *Piscirickettsia* EM genogroup and ten from the LF genogroup were selected for testing and each test was run in five replicates. Resistant strains were distinguished based on the epidemiological cut-off values (ECOFFs). Values specifically determined for *Piscirickettsia* were used for florfenicol (2 µg/ml) and oxytetracycline (4 µg/ml) [19]. Since no ECOFF or MIC break point data for the other antibiotics have so far been determined for *Piscirickettsia*, the MICs for ciprofloxacin (1 µg/ml) and erythromycin (0.5 µg/ml) reported in the EUCAST guidance document for wild type strains of the related *Legionella pneumophila* [20], and the EUCAST ECOFFs for polymyxin B (2 µg/ml), chloramphenicol (8 µg/ml), imipenem (4 µg/ml), ceftazidime (4 µg/ml), gentamycin (2 µg/ml), and ampicillin (8 µg/ml) determined for the *Enterobacterales* were applied [21].

The complete genome nucleotide sequences were uploaded to the Resistance Gene Identifier (RGI) version 5.1.0 of the Comprehensive Antibiotic Resistance Database (CARD) [22], to search for putative resistance conferring genes applying the ‘perfect’ and ’strict’ algorithms which detect known antibiotic resistance proteins and their previously unknown variants, respectively, but no distantly homologous sequences [23]. In order to also search for distant homologs that correlate with, and thus are potentially linked to, the resistance phenotypes, the CARD database version 3.0.7 was downloaded and filtered for the sets of respective resistance genes (e.g., the penam genes for beta-lactam resistance, or 40 different *erm*/*tlr* genes for erythromycin resistance). A BLAST analysis was conducted for all protein sequences of the *Piscirickettsia* genomes with tested resistance phenotype. Results were parsed for hits with e-value ≤1E-30, with minimum identity 30% and minimum query coverage 60%. Additional resistance markers described in literature were also searched for. For the detection of point mutations that resulted in fluoroquinolone resistance, the amino acid sequences of GyrA were aligned using MAFFT v7.313 [refs. 24, 25] with the G-INS-I method and 100 iterative cycles. Similarly, point mutations known to confer resistance were analyzed in penicillin-binding proteins, ribosomal peptides or 23S rRNA sequences, and promotor regions of efflux pumps. The alignments were visualized using the Jalview online tool [26].

**1.6 Phylogeny, digital DNA-DNA Hybridization, Average Nucleotide Identity**

In order to assess the phylogenetic range of the genus *Piscirickettsia*, 16S rRNA gene sequences from all complete genomes were analyzed together with corresponding non-redundant sequences present in the public databases as of January 2020. Thereby, a comprehensive set of all available 238 *Piscirickettsia* 16S rRNA sequences was gathered from the NCBI GenBank database, the scientific literature, and the 73 high quality genomes of the present study. *Piscirickettsia* genomes contain 6 *rrn* operons and in most cases the sequences of all 16S rRNA gene copies within the same genome are identical. Twelve of the 73 analyzed complete genomes contained either one or two 16S rRNA gene sequences with one single nucleotide polymorphism (SNP); these variants were excluded from the phylogenetic analysis. All publicly available 16S rRNA gene sequences designated as *Piscirickettsia salmonis*, *Piscirickettsia* sp., or *Rickettsia*-like organisms, as well as sequences with at least 95% BLASTn identity to the *P. salmonis* type strain 16S rRNA gene sequence were downloaded from NCBI GenBank (Suppl. Table S14). The 16S rRNA gene sequences of *Francisella tularensis* subsp. *tularensis* WY-00W4114 (GenBank CP009753.1), *Francisella tularensis* subsp. *tularensis* SCHU S4 (GenBank AJ749949.2), *Francisella philomiragia* subsp. *philomiragia* ATCC 25017 (GenBank NC_010336.1), *Francisella halioticida* DSM 23729 (GenBank CP022132.1), *Coxiella burnetii* RSA 493 (GenBank NC_002971.4), and *Legionella pneumophila subsp. pneumophila* Philadelphia 1 (GenBank NC_002942.5) served as outgroup. Sequences with lengths of at least 1,300 base pairs were aligned using MAFFT v7.313 [refs. 24, 25] with the G-INS-I method and 100 iterative cycles and the resulting alignment was trimmed to discard leading and trailing base positions not shared by all of the sequences. A phylogenetic tree was reconstructed using RAxML version 8.2.10 [ref. 27] employing algorithm *a* (rapid Bootstrap analysis and search for best-scoring ML tree in one program run), model *GTRCAT* and the number of alternative runs on distinct starting trees was calculated by *autoMR*. Shorter 16S rRNA gene sequences were subsequently inserted without changing the tree topology using the ‘Add marked partial species’ option in ARB [28]. 16S rRNA gene sequence similarity was calculated using the PHYLIP version 3.695 [ref. 29] dnadist tool.

Genome phylogeny was analyzed based on Proteinortho core genome calculations for all complete genomes. For each ortholog group containing exactly one sequence per strain, the corresponding nucleotide sequences from all 73 isolates were extracted and aligned using MAFFT as described above. All alignments were subsequently concatenated using FASconCAT-G v1.04 [ref. 30]. To reconstruct a recombination-free phylogeny, sequence segments identified in the corrected ClonalFrameML analysis (see below) were removed from the concatenated alignment. The genome phylogeny was calculated based on the resulting shortened supermatrix using RAxML employing the same parameters as for the 16S rRNA tree [31]. Due to the very low diversity within the *Piscirickettsia* genogroups and the use of a minimal branch length in RAxML, the branch lengths were recalculated with PhyML v.3.3.3 [refs. 32, 33] for better visualization. Phylogenies that also included draft genomes were calculated using Parsnp v1.2 [ref. 34] with default parameters and the type strain genome ATCC VR-1361 as reference. Phylogenetic trees were drawn using the ggtree R package version 1.16.4 [ref. 35].

Digital DNA-DNA hybridization (dDDH) calculations were performed using the Genome-to-Genome Distance Calculator 2.1 (GGDC) webtool [36] applying the recommended Formula 2 for all pairwise comparisons of complete genomes. Single nucleotide polymorphisms were determined for the complete concatenated core genome alignment employing the snp-dists tool (<https://github.com/tseemann/snp-dists>). For comparison, pairwise Average Nucleotide Identity (ANI) values for closed genomes were calculated using fastANI version 1.0 [ref. 37].

To analyze the phylogeny of the Icm/Dot system core components, the nucleotide sequences of *dotA*, *dotB*, *dotC*, *dotD*, *icmB*, *icmC*, *icmE*, *icmG*, *icmJ*, *icmK*, *icmO*, *icmP*, *icmT*, *icmV*, and *icmW* were collected in homolog groups. The sequences were aligned and alignments concatenated. The phylogeny was calculated using RAxML and visualized using ggtree as described above. The *icm/dot* gene clusters were depicted using EasyFig version 2.2.2 [ref. 38].

**1.7 Chromosome structure, gene order conservation, recombination analysis**

Structural variation of the chromosomes within and between the genogroups was visualized in the Mauve Aligner 2.3.0 (snapshot 2015-02-13, available at <http://darlinglab.org/mauve/snapshots/2015/2015-02-13/>) [39] using default alignment parameters. Gene order conservation was calculated as the fraction of orthologous genes that are syntenous based on at least one shared neighbor (allowing for a gene insertion of one) for all pairwise chromosome comparisons using the synteny-based annotator scripts (<https://github.com/pyelton/Synteny-based-annotator>) [40]. The resulting values were plotted against the average normalized BLAST Bit Score of the respective chromosome pairs. The existing data points for pairwise comparisons between 634 archaeal and bacterial genomes [40] were included and used to calculate a regression based on a linear model and formula y~poly(x, 3), as implemented in the ggplot2 function ‘*geom_smooth’* [41].

To assess the extent of recombination between the *Piscirickettsia* genomes, the nucleotide MAFFT alignments of all 1,530 single-copy core CDSs were concatenated into a supermatrix using FASconCAT-G v1.04 [ref. 30], which yielded an output format suitable for uploading into SplitsTree4 V4.14.6 [ref. 42]. A NeighborNet network was reconstructed applying the ‘UncorrectedP’ and ‘EqualAngle’ options. Recombination events between the same CDSs were additionally identified and quantified applying ClonalFrameML version 1.12 [ref. 43] under a branch-specific mode *(-embranch true*, with standard priors). To distinguish the more recent recombination events, only events that involved nucleotide regions with two or less nucleotide differences between the recipient and a possible donor in another genogroup were considered as recombinations between the different *Piscirickettsia* genogroups [44] whereas all other events were defined as recombinations with genomes outside of the set of 73 genomes. Inspection of the overall 1,884 recombination events identified by ClonalFrame showed that many events are putative false-positives, which occurred where the observed nucleotide diversity was not a result of homologous recombination but likely caused by other sequence modification events or misalignment leading to frameshifts and hence gaps. Some putatively recombining fragments identified were accompanied by a neighboring transposase and contained inverted repeats. In these cases, transposition likely caused the insertion of the fragment rather than classical homologous recombination. The uncorrected number of 1,884 recombination events therefore represents a maximum estimate. All alleged recombining regions that either contained gaps (within the recombining region or up to 50 bp upstream or downstream of it) in the same genome in which the recombination was detected, or which had a transposase as directly neighboring gene, were thus cut from the alignment, the shortened version of the alignment was then again submitted to ClonalFrameML and the program run in a branch-specific mode. This resulted in a corrected (minimum) number of 790 recombination events. For estimating the percentage of the genome subject to recombination, we considered that ClonalFrame may underestimate recombination of small DNA-fragments by a factor of up to ten^43^. The determination of branch-specific r/m values and their 95% confidence intervals were performed as described in issue #114 at <https://github.com/xavierdidelot/ClonalFrameML/issues/114>.

**1.8 Insertion sequence (IS) elements, transposases, extrachromosomal elements**

Transposase ORFs were identified as coding sequences with significant (e-value ≤1E-30, identity ≥30%, query coverage ≥60%) BLAST hits to sequences in the ISfinder database [45] and sorted into established IS families and subgroups in the database by their highest-scoring hit. For comparison, genomes of species closely related to *Piscirickettsia* and of species reported as containing high numbers of IS elements were also included in the analysis. For each of these related bacterial species, four high-quality, closed genomes that were representative for the maximum intraspecific divergence based on the actual ARB-Silva 16S rRNA sequence tree (SILVA_138_SSURef_NR99_05_01_20_opt.arb) were chosen. The strains included in this analysis are listed in Suppl. Table S13.

For phylogenetic analysis and further differentiation of transposase types within established IS family/subgroups, transposase sequences within each family/subgroup were aligned and further split into clusters using the USEARCH v11.0.667 [ref. 46] ‘cluster_fast’ algorithm with a nucleotide identity threshold of 90% to the centroid. The resulting clusters, here termed `transposase types´ were labelled by different capital letters. The threshold was chosen because pairwise divergence values for transposases showed a bimodal frequency distribution with most values falling either well above or below this threshold, and because the threshold is below the divergence of all pairwise comparisons between the *Piscirickettsia* genomes (ANI ≥94,9%; see Results). The individual transposase sequence types thus merge all transposases that likely had a common ancestor. Differences in copy numbers of different transposase types between genomes and genogroups were analyzed by correspondence analysis using vegan::cca (Version 2.5-6) [47] on a matrix with counts of copies of the 106 different transposase types across the 73 genomes.

Transposase types were counted as present in genomes NZ-RLO1, NZ-RLO2 or Y2, if a BLAST hit of the reference sequence with at least 90% identity and 60% query coverage was found. In order to follow the evolution of IS elements during the speciation within *Piscirickettsia*, the Notung-2.9 software package (<http://www.cs.cmu.edu/~durand/Lab/Notung/>) [48] for gene tree-species tree reconciliation was used that supports duplication-loss and duplication-transfer-loss event models with a parsimony-based optimization criterion.

To analyze possible origins of IS elements, reference sequences for each transposase types were blasted against the NCBI GenBank ‘nr’ database excluding all proteins from *Piscirickettsia*genomes, *Piscirickettsiaceae* bacteria NZ-RLO1 and NZ-RLO2, as well as ‘*Piscirickettsia litoralis’*strain Y2.

Changes in the chromosomal location of individual IS elements (i.e., transposition events) were detected through a comparison of the genes flanking individual transposase sequences in the different genomes. For each transposase type in each genogroup, the percentage of stationary transposases was then calculated as the fraction of genomes in which the particular ortholog had identical neighboring genes.

RNA secondary structures in regulatory regions of IS were predicted by submitting the 70 bp-regions upstream of the IS including the start codon to the RNA-fold secondary structure prediction server at <http://rna.tbi.univie.ac.at/cgi-bin/RNAWebSuite/RNAfold.cgi>. Secondary structures with the Minimum Free Energy (MFE) were chosen.

For all extrachromosomal elements (ECEs), pairwise distances between amino acid sequences were calculated and a phylogenetic tree reconstructed using VICTOR with formula D6 [49]. To delineate related ECEs, we identified the most ancestral nodes where all pairwise distances were <33%. The sequence clusters thus defined were extracted from the tree using the R package ggtree [35]. The distance threshold of 33% was chosen based on the following criteria: (i) it was lower than the distance between ECEs occurring in the same strain (in order to coexist in the same cell, ECEs must belong to different compatibility groups; in fact sequence divergence of co-occurring ECEs was ≥51.7% in *Piscirickettsia*), (ii) pairwise distances between ECEs showed a bimodal distribution with a valley at 33% similarity, and (iii) when applying this threshold, all nodes delineating separate ECE clusters were supported with pseudo-bootstrap values of ≥61%. Based on a binary matrix of the presence/absence of the representatives of different ECE clusters, a phylogenetic network of the similarities in ECE content of all *Piscirickettsia* strains was reconstructed by NeighborNet analysis using SplitsTree 4.14.6 [ref. 42].

ECE homologs in other bacteria were searched via BLASTn [50] in the NCBI nucleotide ‘nt’ database [10] (excluding the genus *Piscirickettsia*), in the TARA ocean metagenome database [51], and in the available genomes of the most closely related *Legionellales* [52]. A query coverage above 25% was applied as threshold. In addition, genomes of NZ-RLO1, NZ-RLO2, and Y2 (see above) were analyzed applying a threshold of >50% since the high abundance of similar transposases in *Piscirickettsia* by itself can cause a coverage of up to 49% between nonsyntenous ECEs.

Homologous proteins present in the ECEs were determined with Proteinortho [14] and used to define the core content of each ECE cluster. The quality of specified annotations was checked using UniProt [11, 53]. ECEs were screened for the presence of a type IV secretion system (T4SS) or a characteristic relaxase, which is required for plasmid mobilization [54]. Phage content was analyzed with the PHASTER online tool [55], counting intact, incomplete, as well as questionable hits. All ECEs were also used as query to search for sequence regions of high identity in all *Pisicrickettsia* chromosomes (as subject) by Megablast. Only hits that encompassed ≥90% of the query sequence in up to 3 high scoring pairs were counted.

**1.9 Selection pressure, ancestral state reconstruction, and divergence times**

In order to detect whether positive selection occurred in specific *Piscirickettsia* lineages, the adaptive Branch-Site Random Effects Likelihood method (aBSREL) software [56] was employed for coding sequences of the relaxed core genome (present in at least 95% of genomes) according to the Proteinortho calculations. The translated protein sequences in each family of homologs were first aligned using the MAFFT aligner v7.313 as described above. The resulting alignments were converted into codon alignments employing the PAL2NAL tool [57] and filtered for well aligned conserved regions using Gblocks v. 0.91b [ref. 58]. After deleting duplicate sequences from the filtered nucleotide alignments (keeping note from which genomes the retained unique sequence had originated), those alignments that comprised at least four unique sequences were used for phylogeny reconstruction with RAxML as described above and subsequent aBSREL calculations were run with default parameters. The results were systematically searched for homolog families showing selection on branches representing the *Piscirickettsia* genogroups and subgroups. Since alignment errors (indels) and homologous recombination can also lead to polymorphisms that then will be detected as selection events, we inspected all the alignments for indels and eliminated all false-positive polymorphisms. We also manually checked all of the 200 detected core genes with signs of selection that had evidence for homologous recombination.

To identify codon sites within transposases that evolved under purifying (negative) or diversifying/directional (positive) selection during their expansion (increase in copy number) in a single genome, the Fast Unconstrained Bayesian AppRoximation (FUBAR) method was employed. The amino acid sequences of transposases from each transposase type were aligned using MAFFT and converted to nucleotide alignments using PAL2NAL. The alignments were then split into groups of unique transposase genes from the same chromosome. If these groups consisted of at least four unique sequences, they were used for phylogeny reconstruction with RAxML as detailed above and FUBAR calculations were run with default parameters. Rates of synonymous substitutions (α) and of nonsynonymous substitutions (β) were calculated for each site and a posterior probability > 0.9 was used as significance level to detect sites under negative selection (α > β) or positive selection (α < β) [59]. As FUBAR enables the analysis of all transposase types that occurred in at least four unique copies in the same genome, selective pressure could be analyzed for 46 of the overall 106 transposase types. The copies of single transposase types were aligned and all codon sites analyzed individually. In order to determine to which extent the transposase types that were common in different *Piscirickettsia* genogroups were under different selection pressures, we first compared the copies of a single transposase type in the same genome to determine the codon sites under selection. For each transposase type present in more than one genogroup and for which FUBAR calculations could be performed, the differences in detected selection pressure between genogroups were calculated. For this, the percentages of genomes in which each site was found to be under selection in the *Piscirickettsia* genogroup in which the transposase type had the highest copy number were determined. Calculations were done separately for sites under positive or negative selection. The percentage values for all the sites in one transposase type were then concatenated to yield one numerical vector (as an R data structure type). The same procedure was followed to determine the percentages of genomes of all other genogroups in which each site was found to be under selection. As a measure of the pairwise differences in the selection pressure on each transposase cluster between genogroups, the Euclidian distance between the two corresponding numerical vectors was calculated. The Euclidian distance value was visualized as the area of pie charts for which the distance values were summed up for positive and negative selection and depicted as separate sectors.

BadiRate [60] was employed to analyze gene gains and losses. For each of the homolog groups identified in the Proteinortho analysis, one reference sequence was uploaded to the eggNOG-mapper v2 [ref. 61] using DIAMOND search [62] and eggNOG v5.0 clusters and phylogenies [63]. The Proteinortho output matrix was transformed to a new numeric matrix and split according to the COG categories assigned by eggNOG. From the resulting matrices, those containing homolog groups that had been assigned exactly one COG group were used as input for BadiRate analyses. These analyses were run with the RAxML core genome tree, after transformation to an ultrametric tree using the R 'ape' package [64, 65], estimation procedure Maximum Likelihood, BDI family turnover rates under the free rates model and with parameters 'anc' and 'outlier'.

Ancestral character state reconstructions of the number of genes per homolog groups, the number of pseudogenes per homolog group, and the number of extrachromosomal elements under the Maximum Parsimony Criterion were conducted using the MPR function of the 'ape' R package [64]. The function was chosen because it supported all observed character states. The numbers of changes per branch were summed up separately for transposases and other genes.

BayesTraits V3.0.2 was used to apply the continuous regression model and independent contrasts [65, 66] under the maximum likelihood criterion between log-transformed gene and pseudogene counts to test the effect of the number of transposase genes on overall gene and pseudogene content. BayesTraits V3.0.2 considers the effect of phylogenetic distance on gene gains and losses. Phylogeny-unaware regression would be inappropriate for these types of data [67, 68]. The log transformation was necessary because count data of this magnitude cannot be modelled as discrete multi-state characters in the program. (Results from continuous regression model and the simpler independent contrasts approach are almost identical).

The software BEAST v2.6.3 [ref. 69] was used to compute divergence times based on the recombination-corrected core genome alignment previously used to construct a RAxML phylogeny. Estimations of evolutionary rates were attempted with 10^8^ iterations after a burn-in of 10% with both, strict and relaxed clock models, and tree priors for constantly or exponentially growing population sizes, respectively and combining three independent runs. A date-randomization test, in which a substitution-rate estimate had to fall outside the 95% credibility intervals of rates that were estimated after randomizing sampling times among sequences [70, 71] failed, indicating that the temporal signal in the available *Piscirickettsia* data was insufficient for calibrating the molecular clock. We therefore applied a previously reported base substitution rate from *Legionella pneumophila* [72] as a fixed clock rate to estimate evolutionary divergence times within *Piscirickettsia*. For these computations, 10^7^ iterations (with 10% burn-in) were sufficient.

**1.10 Biogeographic and temporal patterns, and host distribution of *Piscirickettsia***

All metadata available for *Piscirickettsia* phylotypes and genomes were collected, including the geographic origin, year of isolation, and host species (listed in Suppl. Tables S1 and S12). Tip-permutation tests were performed to determine the probability of the null hypothesis that the three character states observed for individual phylotypes or genomes are distributed randomly with respect to the topology of the corresponding phylogenetic tree [73]. The parsimony scores and the other indexes were calculated with PAUP* [74]. The tips of the tree were permuted randomly and the frequency of those of the permuted trees for which character distribution yielded a parsimony score at least as low as that of the original tree was recorded. Results are given as *p*-values of the permutation test (*p*-values ≤0.001 were recorded as significant) and as the retention index RI. The latter represents a parsimony score that is corrected for the range of scores of the particular character and is bound between 0 and 1, with 1 indicating a perfect fit.

All amino acid sequences of representative genomes of the three genogroups (ATCC VR-1361 for LF, Psal-002 for EM, MR5 and NVI 5692 for the Canadian genomes and the Norwegian genomes, respectively) were blasted against the MGnify protein database [75]. The results were filtered for at least 95% identity (based on the variability of amino acid sequences within individual genogroups), a length of >30 amino acids, as well as a maximum e-value of 1E-30. For each MGnify protein producing a hit meeting these criteria, the corresponding analysis accession number was extracted from the ‘mgy_assemblies.tsv.gz’ file and the geographic coordinates for the sample origin were retrieved from the European Nucleotide Archive (ENA) website at https://www.ebi.ac.uk/ena/browser/home.

**2. SUPPLEMENTARY RESULTS**

**2.1 Comparison of genome sizes of *Piscirickettsia* with those of related intracellular *Legionellales***

The shortest genomes in the *Legionella* genus (of species *L. adelaidensis*, *geestiana*, *londiniensis*, *oakridgensis*) are just 2.37-2.55 Mb [76], those of *Coxiella burnetii* strains are 1.99-2.03 Mb [77] long, the genome of *Aquicella siphonis* (NCBI Genome ID 83687) has 2.83 Mb, that of *A. lusitana* (NCBI Genome ID 71313) 2.58 Mb, and that of *Francisella* spp. 1.70-2.24 Mb [78].

**2.2 Evidence for the delineation of the three *Piscirickettsia* genogroups as separate species**

The distinct phylogenomic clustering of the available *Piscirickettsia* genomes is corroborated by three additional analyses. Employing the Type (Strain) Genome Server (TYGS [79]), the EM genogroup has to be considered a separate species whereas the NC genogroup would represent a distinct subspecies within a second species composed of both the NC and LF genogroups. Pairwise Average Nucleotide Identity (ANI) ranged from 94.9% to 97.5% for pairwise comparisons between the three genogroups (Suppl. Fig. S3A). The Genome Taxonomy Database (GTDB) uses the same tool as the one applied in our study for ANI calculations, but employs a conservative species threshold of 95% and, as a result, groups all *P. salmonis* genomes into a single species [80]. However, the ANI values actually fell into the ANI transition zone for species demarcation of 95-96% that was proposed by [81]. Applying this range, the three genogroups can be delineated as distinct species. Also, Digital DNA-DNA hybridization (dDDH) computations yielded values below the species demarcation threshold for the pairwise comparisons LF-EM and EM-NC (Suppl. Fig. S3B).

**2.3 Mechanisms of genetic separation of *Piscirickettsia* genogroups and EM subgroups**

Of the 790 to 1,868 recombination events (minimum-maximum estimates, see Materials and Methods), just 29 represented homologous recombination events between the three genogroups. The mean length of recombining fragments was 74 bp. Even when considering that ClonalFrameML may underestimate the number of homologous recombination events for short fragments by a factor of up to ten^43^, recombination affected only a minor fraction (<3.5%) of the *Piscirickettsia* genomes.

Our comparative genome analyses provided evidence for the existence of genetic barriers to homologous recombination that involved genogroup-specific Restriction-Modification (R-M) systems as well as disruptions in competence genes (Suppl. Table S3A). The three Canadian genomes carry homologs of *hsdM*, *hsdS*, and *hsdR* which encode a type IC R-M system most closely related to *Eco*R124II and which are arranged in the canonical order. Whereas HsdM and HsdR had high amino acid sequence similarity to the *Eco*R124II homologs (85 and 75%, respectively), similarity was only 37% for HsdS. Since HsdS determines the target sequence specificity, its lower sequence identity likely results in the recognition of a different sequence motif for methylation. Indeed, our SMRT sequencing provided independent support for the activity of the type I R-M system and an alternative methylation target sequence: the asymmetric bipartite nucleotide sequence motif CG**^6m^A**N_7_TTGG occurred 230 times in the genomes of Canadian strains and over 98% contained 6-methyl adenosine on both strands (Suppl. Table S3B). This motif resembles, but is not identical to, known enterobacterial type I R-M target sequences [82]. The genome of the Norwegian strain NVI 5692 completely lacks methylation and a cognate R-M system, which very likely contributes towards the genetic isolation of the Canadian from the Norwegian strains [83]. While additional R-M systems were detected in the other genomes, their role cannot be inferred at present (Suppl. Table S3B).

Insertional inactivation of the competence genes was the second mechanism of genetic isolation that could be readily detected in all genomes. ComEC is the essential channel protein for DNA binding and uptake, and showed disruptions by insertion sequence (IS) elements which were localized in a consistent genogroup- and even subgroup-specific pattern (Suppl. Fig. S6, Suppl. Table S3C). In addition, the gene *comF*, encoding a phosphoribosyltransferase interacting with the channel protein, was disrupted by an identical frameshift in the Canadian strains and the type strain ATCC VR-1361 (Suppl. Table S3D). The largely group-specific transposition and frameshift patterns indicate that the inactivation of the competence system occurred independently in the different *Piscirikettsia* genogroups but mostly prior to their subsequent within-group diversification.

**2.4 Extrachromosomal elements and antibiotic resistance of *Piscirickettsia***

The isolates carried up to seven extrachromosomal elements (ECEs) with most of the genomes (i.e., 53) containing four ECEs. Overall, 290 ECEs with sizes between 9,266 and 251,458 bp were detected (Suppl. Table S4A). Most ECEs represented low copy number plasmids (median, 1.9 per genome) based on observed Illumina read coverages with one possible exception (ECE type 2). 286 ECEs did not show any similarity to available plasmid sequences in other bacterial genera. Plasmid incompatibility groups are not yet defined for *Piscirickettsia* because of the absence of recognizable replication systems [84]. We therefore delineated ECE groups based on the co-occurrence, sequence similarity, and phylogenetic relationships of the ECEs (see Suppl. Information 1.8), and could distinguish 23 groups of closely related ECEs plus 19 singletons (Suppl. Fig. S7). The distribution of ECE types was strictly genogroup-specific and even EM subgroup-specific, and each of the 23 ECEs with multiple occurrences occurred in most of the representatives of a genogroup or EM subgroups (median, 91%). ECE type 6 occurred in all four EM subgroups, and likely resulted from a phage transduction event (Suppl. Table S4A). A NeighborNet analysis of the distribution of all ECE types over the genomes showed a clear genetic separation of the three genogroups and a separation of single EM subgroup, similar to the core genome network (Fig. 1H, Suppl. Fig. 4C). Plasmid transfer therefore has been a rare event between the three *Piscirickettsia* genogroups and was also limited between the different EM subgroups. The 286 *Piscirickettsia*-specific ECEs had a GC content very similar to the chromosome (36.4 - 40.7 mol%; compare Suppl. Table S2). Two thirds of the ECEs contained toxin-antitoxin systems, which likely facilitate their stable maintenance [85]. Only 4 of the 42 ECE types contained type IV secretion-like systems indicating mobility of these ECEs (Suppl. Table S4A). Collectively, our data indicate an early acquisition of most ECEs by the ancestors of the different genogroups or EM subgroups and an efficient maintenance of ECEs during subsequent diversification. Sequences of ECEs 11 and 42 could even be detected in the more distant, but incomplete, genomes of NZ-RLO1 and Y2, pointing to an even earlier acquisition of some ECEs.

A previously described resistance plasmid and its derivative [86] were found in only four of the 73 strains. Most likely, the antibiotic resistance plasmid ECE 5 was acquired only once and remained in this clonal and local subpopulation, followed by gene losses that yielded ECE 7 in one of the isolates (AY6532B). The rare occurrence of horizontally acquired resistance genes may suggest that the emergence of antibiotic resistance in *Piscirickettsia* may be retarded compared to other salmon pathogens such as *Renibacterium salmoninarum* [87, 88]. Instead, the long persistence of ECEs within entire genogroups and the almost complete lack of typical antibiotic resistance markers indicate that the majority of ECEs confer other selective advantages to the pathogen. Similarity searches against the Virulence Factors Database (VFDB) identified putative virulence factors in 177 of the 290 ECEs. Virulence factors occurred in similar numbers in ECEs of the same type (Suppl. Table S4A). Recently, eleven of these putative virulence factors were shown to be overexpressed in strain ATCC VR-1361 after infection of two different cells lines [84], pointing to a role of ECEs in host adaptation.

All *Piscirickettsia* strains showed a so far undocumented resistance to erythromycin, gentamicin, and polymyxin. Resistance to imipenem, ampicillin, and ceftazidime was highly variable with MIC values varying up to thousandfold between strains. These resistances likely are intrinsic since we found 30 orthologues of efflux pump components that might be involved in gentamycin and beta-lactam resistances and also detected two point mutations which are known to cause intrinsic polymyxin resistance in other bacteria (Suppl. Tables S5, S7). Indeed, different cellular efflux rates and induction patterns of RND-type *acrAB*-homologs have already been documented in two *Piscirickettsia* strains [89]. In contrast, we did not detect any specific resistance genes against the six antibiotics on plasmids.

**2.5 Transposases of *Piscirickettsia***

The transposases fell into 20 different insertion sequence (IS) families/subgroups which showed distinct abundance patterns across the three *Piscirickettsia* genogroups (Suppl. Fig. S9). Since single IS families can encompass diverse transposases [90, 91], we assessed the intrafamily relatedness of transposases, and discovered up to 12 phylogenetically distinct sequence clusters within single IS families. The different clusters (here denoted `transposase types´ and distinguished using capital letters) of the same IS family not only were separated by their low sequence similarities but were also distributed differently across the genogroups (Suppl. Table S8), suggesting a distinct biology. In total, *Piscirickettsia* harbors an unprecedented diversity of 106 transposase types of which 61 were confined to single genogroups whereas only 12 were common to all three (insert, Suppl. Fig. S9). Within the EM genogroup, 15 of the 41 transposase types even occurred only in particular subgroups, corroborating the genetic distinctness of the EM subgroups (Suppl. Table S8). Over two thirds of the transposase types were present in multiple copies in each *Piscirickettsia* genome; 13 occurred in >20 copies, with up to 248 copies in the Canadian strains (Suppl. Table S8). For some of these IS elements, bursts of transposition have previously been documented in *E. coli* (e.g., IS*30* [ref. 92]).

Genome sequencing revealed independent evidence for ongoing transposition events. The genomic regions around an *icm/dot* gene cluster in EM strain Psal-072 and LF strain Psal-073 showed twice the read coverage of the remaining chromosome, which points to extrachromosomal duplication of these regions (as ECEs Psal-072_CP039041 and Psal-073_CP039048) (Suppl. Fig. S10). The excision sites were identified close to IS*30*_A or IS*982*_C type transposases. Copied and excised chromosomal regions were also recognized in two other LF strains (ECEs Psal-006b_CP038900, Psal-013_CP038931; Suppl. Tables S4A,B,C).

Expansion of several transposases in the LF (IS*30*_A, IS*4_*IS*4*_A, IS*982*_A, IS*3_*IS*3*_A) and NC genogroups (IS*630*_B, IS*30*_B, IS*630_*C, IS*701_*IS*Aba11*_C) must have been accompanied much more often by transposase immobilization than the expansion of transposases in the EM genogroup (Fig. 3A). Eventually, continued inactivation and accompanying losses result in the immobilization of transposases and a decrease of their copy numbers during genome evolution [93, 94]. We found a higher fraction of transposases in LF and NC-genogroups to be truncated than in the EM genogroup (Suppl. Table S9A, see below), corroborating a progressed state of inactivation in the first two genogroups. In the EM genogroup, however, particularly the high copy number transposases IS*630*_A, IS*701_*IS*Aba11*_A, IS*30*_A, IS*6*_A retained their high mobility during expansion. Transposase activity may also be attenuated through the repression of translation by formation of stable hairpin structures of transcripts as previously described for IS*200* in other Gammaproteobacteria [95]. Indeed, 80% of the IS*200_A* transposases in *Piscirickettsia* were stationary and an analysis of upstream sequence regions using the RNAfold software predicted potentially stable secondary mRNA structures at the AGATTA ribosome binding site. Similarly, hairpin formation of IS transcripts was also predicted for some other low copy number transposases (IS*3_*IS*3_*L, IS*30_*I, IS*6_*F and IS*As1_*B), suggesting that attenuation occurs also in other transposase families.

The analyzed transposase types (see Materials and Methods) had between up to 39 codon sites under purifying (negative) selection and up to six under diversifying (positive) selection in at least one *Piscirickettsia* genome (Suppl. Table S8). In the EM genogroup, a larger number of transposase types exhibited signatures of selection than in the LF genogroup (20 versus 15). The highest number of sites under selection was detected in transposase type IS*6*_A. Pronounced differences in selection pressure were even detected between the EM subgroups for several transposase types (data not shown), indicating that transposases were also involved in the evolutionary split of the EM genogroup.

Our analysis of pseudogenes revealed that truncation represents the dominant mechanism of pseudogenization in *Piscirickettsia* and resulted in the formation of 380-964 putative pseudogenes (12-20% of all ORFs; Suppl. Table S9A). Between 5.4 and 15.4% of all transposases were truncated, representing less than one tenth of all truncated genes. In addition, 124 to 209 recognizable gene fragments per genome were detected in intergenic regions. Genomes of the EM genogroup had the lowest numbers and percentages of pseudogenes, followed by the LF genogroup and the Norwegian genome. The genomes of the Canadian subgroup showed the highest numbers and percentages of pseudogenization. While a random distribution across genomes would result in 26-42% of pseudogenes being flanked by transposases, the actual fraction of flanking transposases was higher for pseudogenized CDSs (43-59%), for pseudogenized transposases (up to 46-58%), as well as intergenic pseudogene fragments (75-87%) (Suppl. Fig. S13, Suppl. Table. S9B).

The level of pseudogenization detected in *Piscirickettsia* by far exceeds that of most other prokaryotic genomes (1-5% of all CDSs [96]), even that of *Shigella flexneri* which is in an intermediate stage of host adaptation (10% [ref. 18]) and is only surpassed by those of obligate intracellular pathogens or symbionts (37-44% pseudogenes [96–98]).

**2.6 Genogroup-specific gene content**

Based on the distribution of homolog groups across the genomes (Suppl. Fig. S14), 1,767 homolog groups were present in all genomes, constituting the core genome, and 1,293 coding sequences occurred only in single genomes. Specific gene inventories were also observed for the 3, 9, 33, and 36 genomes of the different genogroups or subgroups (arrows in Suppl. Fig. S14). Larger fractions of genogroup-specific genes were also apparent for two Canadian genomes and 32 LF genomes, likely due to the loss of common homologs in single members of the genogroup; these homolog groups were therefore also included in the subsequent analysis of specific gene inventories (Suppl. Table S10).

The pseudaminic acid biosynthesis pathway was present in all *Piscirickettsia* isolates except the Canadian strains and had a structure similar to that in *Acinetobacter baumannii*, containing orthologs of *pseB*, *pseC*, *pseF*, *pseI*, but differing by a fusion of the *pseG* and *pseH* genes. Pseudaminic acid is found in the capsule polysaccharide of *A. baumannii* [99], has been linked to virulence in several bacterial species [100, 101], and has experimentally been shown to be a constituent of the lipopolysaccharide of *Piscirickettsia* [102].

Aside from the genes involved in differentiation of cell surfaces described in the main text, other group-specific genes were identified and involve the exclusive presence of phosphate acetyltransferase and acetate kinase (groups 3197, 3198; Suppl. Table S10) in the Canadian strains, five different peptidase-like genes that occurred exclusively, or were positively selected, in the NC genogroup (group 2618 unique to the entire NC genogroup, group 138 unique to the Canadian strains, and groups 5682, 5822 are unique and group 185 was positively selected in the Norwegian strain), as well as six and two signature genes of defense systems in the Norwegian and Canadian genomes, respectively. In addition, 11 genes encoding transporter proteins also showed distinct distribution patterns across *Piscirickettsia*. Six of them, annotated as specific glucose permease (*glcU*), and transporters of arsenite (*arsB*), prolin, malonate, and polar amino acids, as well as a homolog to outer membrane protein MipA (groups 2758, 3020, 3225, 3286, 3349, 3414, respectively) were limited to Canadian strains. Two genes were specific for the Norwegian strain (groups 3809, 5554), three were specific for the entire NC genogroup (groups 622, 2776, 2792), one was specific for the LF genogroup (group 4885), and an oligopeptide transporter was under positive selection only in the EM genogroup (group 210) (Suppl. Table S10). We hypothesize that this specific distribution patterns of transporters confers a selective advantage in the presence of particular carbon or inhibiting substrates. Finally, genes involved in purin/pyrimidine (7 genes), amino acid synthesis pathways (3 genes), chemotaxis/signal transduction (5 genes), or secretion (4 genes), as well as 23 genes involved in DNA replication/repair and transcription were differently distributed across the genogroups. While the potential role of these latter genes in adaptation is presently unclear, their distribution indicates that *Piscirickettsia* genogroups may have also differentiated with respect to central metabolic processes and genetic information processing.

The unusual occurrence of several *icm/dot* gene clusters could render *Piscirickettsia* more resistant to mutational inactivation. However, it has been shown previously that two of the *icm/dot* clusters are not functionally interchangeable and may be important during different stages of infection, since mutational inactivation of *icmB* of one of the gene clusters attenuated pathogenicity without any evidence for complementation by the intact second *icm/dot* system [103]. We detected single insertions and deletions in *icm/dot* genes resulting in frameshifts in 11 different *Piscirickettsia* strains and even insertion events of one 288bp-long transposase fragment (IS*982*_A; Suppl. Table S8) in four strains (Suppl. Table S11B). More sites of *icm/dot* clusters A and B were mutated in the LF genomes and these signs of gene decay were more prominent for cluster A than for cluster B. Ten different sites of *icm/dot* cluster A were mutated in the LF genomes but only four sites in EM genomes. In contrast, no mutation could be detected in cluster C of the LF strains and in the Norwegian strain. It is presently unknown at which stage of infection the third *icm/dot* cluster is relevant and whether its expression could then compensate for an inactivation of cluster A or B in the LF genomes. The genogroup-specific differences in the number of *icm/dot* gene clusters and their different degree of mutational inactivation may at least partly explain the differences in virulence observed for different *Piscirickettsia* strains.

**References:**

1. Contreras-Lynch S, Smith P, Olmos P, Loy ME, Finnegan W, Miranda CD. A novel and validated protocol for performing MIC tests to determine the susceptibility of *Piscirickettsia salmonis* isolates to florfenicol and oxytetracycline. *Front Microbiol* 2017; **8**: 1255.

2. Yañez AJ, Silva H, Valenzuela K, Pontigo JP, Godoy M, Troncoso J, et al. Two novel blood-free solid media for the culture of the salmonid pathogen *Piscirickettsia salmonis*. *J Fish Dis* 2013; **36**: 587–591.

3. Yañez A, Valenzuela K, Silva H, Retamales J, Romero A, Enriquez R, et al. Broth medium for the successful culture of the fish pathogen *Piscirickettsia salmonis*. *Dis Aquat Organ* 2012; **97**: 197–205.

4. Mandakovic D, Glasner B, Maldonado J, Aravena P, González M, Cambiazo V, et al. Genomic-based restriction enzyme selection for specific detection of *Piscirickettsia salmonis* by 16S rDNA PCR-RFLP. *Front Microbiol* 2016; **7**: 643.

5. Marshall S, Heath S, Henríquez V, Orrego C. Minimally invasive detection of *Piscirickettsia salmonis* in cultivated salmonids via the PCR. *Appl Environ Microbiol* 1998; **64**: 3066–3069.

6. Contreras-Lynch S, Olmos P, Vargas A, Figueroa J, González-Stegmaier R, Enríquez R, et al. Identification and genetic characterization of *Piscirickettsia salmonis* in native fish from southern Chile. *Dis Aquat Organ* 2015; **115**: 233–244.

7. Li H, Durbin R. Fast and accurate short read alignment with Burrows-Wheeler transform. *Bioinformatics* 2009; **25**: 1754–1760.

8. Koboldt DC, Zhang Q, Larson DE, Shen D, McLellan MD, Lin L, et al. VarScan 2: somatic mutation and copy number alteration discovery in cancer by exome sequencing. *Genome Res* 2012; **22**: 568–576.

9. Seemann T. Prokka: rapid prokaryotic genome annotation. *Bioinformatics* 2014; **30**: 2068–2069.

10. Benson DA, Cavanaugh M, Clark K, Karsch-Mizrachi I, Ostell J, Pruitt KD, et al. GenBank. *Nucleic Acids Res* 2018; **46**: D41–D47.

11. Boutet E, Lieberherr D, Tognolli M, Schneider M, Bansal P, Bridge AJ, et al. UniProtKB/Swiss-Prot, the manually annotated section of the UniProt KnowledgeBase: how to use the entry view. In: Edwards D (ed). *Plant Bioinformatics. Methods in Molecular Biology*. 2016. Humana Press, New York, NY, pp 23–54.

12. Altschul SF, Gish W, Miller W, Myers EW, Lipman DJ. Basic local alignment search tool. *J Mol Biol* 1990; **215**: 403–410.

13. Kanehisa M, Sato Y, Morishima K. BlastKOALA and GhostKOALA: KEGG tools for functional characterization of genome and metagenome sequences. *J Mol Biol* 2016; **428**: 726–731.

14. Lechner M, Findeiß S, Steiner L, Marz M, Stadler PF, Prohaska SJ. Proteinortho: detection of (co-)orthologs in large-scale analysis. *BMC Bioinformatics* 2011; **12**: 124.

15. Chen L. VFDB: a reference database for bacterial virulence factors. *Nucleic Acids Res* 2004; **33**: D325–D328.

16. Liu B, Zheng D, Jin Q, Chen L, Yang J. VFDB 2019: a comparative pathogenomic platform with an interactive web interface. *Nucleic Acids Res* 2019; **47**: D687–D692.

17. Roberts RJ, Vincze T, Posfai J, Macelis D. REBASE—a database for DNA restriction and modification: enzymes, genes and genomes. *Nucleic Acids Res* 2015; **43**: D298–D299.

18. Lerat E, Ochman H. Ψ-Φ: exploring the outer limits of bacterial pseudogenes. *Genome Res* 2004; **14**: 2273–2278.

19. Henríquez P, Kaiser M, Bohle H, Bustos P, Mancilla M. Comprehensive antibiotic susceptibility profiling of Chilean *Piscirickettsia salmonis* field isolates. *J Fish Dis* 2016; **39**: 441–448.

20. The European Committee on Antimicrobial Susceptibility (EUCAST). Guidance document on antimicrobial susceptibility testing of Legionella pneumophila - May 2021. https://www.eucast.org/fileadmin/src/media/PDFs/EUCAST_files/Guidance_documents/Legionella_guidance_note_-_20210528.pdf. Accessed 20 Jul 2021.

21. The European Committee on Antimicrobial Susceptibility (EUCAST). Breakpoint tables for interpretation of MICs and zone diameters. Version 10.0, 2020. https://www.eucast.org/fileadmin/src/media/PDFs/EUCAST_files/Breakpoint_tables/v_10.0_Breakpoint_Tables.pdf. Accessed 22 Oct 2020.

22. Jia B, Raphenya AR, Alcock B, Waglechner N, Guo P, Tsang KK, et al. CARD 2017: expansion and model-centric curation of the comprehensive antibiotic resistance database. *Nucleic Acids Res* 2017; **45**: D566–D573.

23. Alcock BP, Raphenya AR, Lau TTY, Tsang KK, Bouchard M, Edalatmand A, et al. CARD 2020: antibiotic resistome surveillance with the comprehensive antibiotic resistance database. *Nucleic Acids Res* 2019; **48**: D517–D525.

24. Katoh K. MAFFT: a novel method for rapid multiple sequence alignment based on fast Fourier transform. *Nucleic Acids Res* 2002; **30**: 3059–3066.

25. Katoh K, Standley DM. MAFFT multiple sequence alignment software version 7: improvements in performance and usability. *Mol Biol Evol* 2013; **30**: 772–780.

26. Waterhouse AM, Procter JB, Martin DMA, Clamp M, Barton GJ. Jalview Version 2 - a multiple sequence alignment editor and analysis workbench. *Bioinformatics* 2009; **25**: 1189–1191.

27. Stamatakis A. RAxML version 8: a tool for phylogenetic analysis and post-analysis of large phylogenies. *Bioinformatics* 2014; **30**: 1312–1313.

28. Ludwig W, Klenk H. Overview: A Phylogenetic Backbone and Taxonomic Framework for Procaryotic Systematics. *Bergey’s Manual of Systematics of Archaea and Bacteria*. 2015. Wiley, pp 1–27.

29. Felsenstein J. PHYLIP: Phylogeny Inference Package. http://evolution.genetics.washington.edu/phylip.html. Accessed 21 Jul 2022.

30. Kück P, Longo GC. FASconCAT-G: extensive functions for multiple sequence alignment preparations concerning phylogenetic studies. *Front Zool* 2014; **11**: 81.

31. Hahnke RL, Meier-Kolthoff JP, García-López M, Mukherjee S, Huntemann M, Ivanova NN, et al. Genome-based taxonomic classification of *Bacteroidetes*. *Front Microbiol* 2016; **7**: 2003.

32. Guindon S, Delsuc F, Dufayard J-F, Gascuel O. Estimating maximum likelihood phylogenies with PhyML. In: Posada D (ed). *Bioinformatics for DNA Sequence Analysis. Methods in Molecular Biology*. 2009. Humana Press, New York, NY, pp 113–137.

33. Guindon S, Dufayard J-F, Lefort V, Anisimova M, Hordijk W, Gascuel O. New algorithms and methods to estimate maximum-likelihood phylogenies: assessing the performance of PhyML 3.0. *Syst Biol* 2010; **59**: 307–321.

34. Treangen TJ, Ondov BD, Koren S, Phillippy AM. The Harvest suite for rapid core-genome alignment and visualization of thousands of intraspecific microbial genomes. *Genome Biol* 2014; **15**: 524.

35. Yu G. Using ggtree to visualize data on tree-like structures. *Curr Protoc Bioinformatics* 2020; **69**: e96.

36. Meier-Kolthoff JP, Auch AF, Klenk H-P, Göker M. Genome sequence-based species delimitation with confidence intervals and improved distance functions. *BMC Bioinformatics* 2013; **14**: 60.

37. Jain C, Rodriguez-R LM, Phillippy AM, Konstantinidis KT, Aluru S. High throughput ANI analysis of 90K prokaryotic genomes reveals clear species boundaries. *Nat Commun* 2018; **9**: 5114.

38. Sullivan MJ, Petty NK, Beatson SA. Easyfig: a genome comparison visualizer. *Bioinformatics* 2011; **27**: 1009–1010.

39. Darling ACE, Mau B, Blattner FR, Perna NT. Mauve: multiple alignment of conserved genomic sequence with rearrangements. *Genome Res* 2004; **14**: 1394–1403.

40. Yelton AP, Thomas BC, Simmons SL, Wilmes P, Zemla A, Thelen MP, et al. A semi-quantitative, synteny-based method to improve functional predictions for hypothetical and poorly annotated bacterial and archaeal genes. *PLoS Comput Biol* 2011; **7**: e1002230.

41. Wickham H. ggplot2. 2016. Springer International Publishing, Cham.

42. Huson DH, Bryant D. Application of phylogenetic networks in evolutionary studies. *Mol Biol Evol* 2006; **23**: 254–267.

43. Didelot X, Wilson DJ. ClonalFrameML: efficient inference of recombination in whole bacterial genomes. *PLoS Comput Biol* 2015; **11**: e1004041.

44. Didelot X, Barker M, Falush D, Priest FG. Evolution of pathogenicity in the *Bacillus cereus* group. *Syst Appl Microbiol* 2009; **32**: 81–90.

45. Siguier P. ISfinder: the reference centre for bacterial insertion sequences. *Nucleic Acids Res* 2006; **34**: D32–D36.

46. Edgar RC. Search and clustering orders of magnitude faster than BLAST. *Bioinformatics* 2010; **26**: 2460–2461.

47. Oksanen J, Blanchet FG, Friendly M, Kindt R, Legendre P, McGlinn D, et al. vegan: Community Ecology Package. R package version 2.5-6. http://CRAN.R-project.org/package=vegan. Accessed 27 May 2019.

48. Chen K, Durand D, Farach-Colton M. NOTUNG: A program for dating gene duplications and optimizing gene family trees. *Journal of Computational Biology* 2000; **7**: 429–447.

49. Meier-Kolthoff JP, Göker M. VICTOR: genome-based phylogeny and classification of prokaryotic viruses. *Bioinformatics* 2017; **33**: 3396–3404.

50. Ye J, McGinnis S, Madden TL. BLAST: improvements for better sequence analysis. *Nucleic Acids Res* 2006; **34**: W6–W9.

51. Sunagawa S, Coelho LP, Chaffron S, Kultima JR, Labadie K, Salazar G, et al. Structure and function of the global ocean microbiome. *Science (1979)* 2015; **348**: 1261359.

52. Hugoson E, Guliaev A, Ammunét T, Guy L. Host Adaptation in *Legionellales* Is 1.9 Ga, Coincident with Eukaryogenesis. *Mol Biol Evol* 2022; **39**: msac037.

53. The UniProt Consortium. UniProt: a worldwide hub of protein knowledge. *Nucleic Acids Res* 2019; **47**: D506–D515.

54. Smillie C, Garcillán-Barcia MP, Francia MV, Rocha EPC, de la Cruz F. Mobility of plasmids. *Microbiology and Molecular Biology Reviews* 2010; **74**: 434–452.

55. Arndt D, Grant JR, Marcu A, Sajed T, Pon A, Liang Y, et al. PHASTER: a better, faster version of the PHAST phage search tool. *Nucleic Acids Res* 2016; **44**: W16–W21.

56. Smith MD, Wertheim JO, Weaver S, Murrell B, Scheffler K, Kosakovsky Pond SL. Less is more: an adaptive branch-site random effects model for efficient detection of episodic diversifying selection. *Mol Biol Evol* 2015; **32**: 1342–1353.

57. Suyama M, Torrents D, Bork P. PAL2NAL: Robust conversion of protein sequence alignments into the corresponding codon alignments. *Nucleic Acids Res* 2006; **34**: W609–W612.

58. Talavera G, Castresana J. Improvement of phylogenies after removing divergent and ambiguously aligned blocks from protein sequence alignments. *Syst Biol* 2007; **56**: 564–577.

59. Murrell B, Moola S, Mabona A, Weighill T, Sheward D, Kosakovsky Pond SL, et al. FUBAR: a Fast, Unconstrained Bayesian AppRoximation for inferring selection. *Mol Biol Evol* 2013; **30**: 1196–1205.

60. Librado P, Vieira FG, Rozas J. BadiRate: estimating family turnover rates by likelihood-based methods. *Bioinformatics* 2012; **28**: 279–281.

61. Cantalapiedra CP, Hern̗andez-Plaza A, Letunic I, Bork P, Huerta-Cepas J. eggNOG-mapper v2: functional annotation, orthology assignments, and domain prediction at the metagenomic scale. *Mol Biol Evol* 2021; **38**: 5825–5829.

62. Buchfink B, Reuter K, Drost HG. Sensitive protein alignments at tree-of-life scale using DIAMOND. *Nat Methods* 2021; **18**: 366–368.

63. Huerta-Cepas J, Szklarczyk D, Heller D, Hernández-Plaza A, Forslund SK, Cook H, et al. EggNOG 5.0: a hierarchical, functionally and phylogenetically annotated orthology resource based on 5090 organisms and 2502 viruses. *Nucleic Acids Res* 2019; **47**: D309–D314.

64. Paradis E, Schliep K. ape 5.0: an environment for modern phylogenetics and evolutionary analyses in R. *Bioinformatics* 2019; **35**: 526–528.

65. Felsenstein J. Maximum likelihood estimation of evolutionary trees from continuous characters. *Am J Hum Genet* 1973; **25**: 471–492.

66. Freckleton RP. Fast likelihood calculations for comparative analyses. *Methods Ecol Evol* 2012; **3**: 940–947.

67. Harvey PH, Pagel MD. The Comparative Method in Evolutionary Biology. 1991. Oxford University Press, Oxford.

68. Felsenstein J. Phylogenies and the comparative method. *American Naturalist* 1985; **125**: 1–15.

69. Bouckaert R, Vaughan TG, Barido-Sottani J, Duchêne S, Fourment M, Gavryushkina A, et al. BEAST 2.5: An advanced software platform for Bayesian evolutionary analysis. *PLoS Comput Biol* 2019; **15**: e1006650.

70. Duchêne S, Duchêne D, Holmes EC, Ho SYW. The performance of the date-randomization test in phylogenetic analyses of time-structured virus data. *Mol Biol Evol* 2015; **32**: 1895–1906.

71. Nübel U, Dordel J, Kurt K, Strommenger B, Westh H, Shukla SK, et al. A timescale for evolution, population expansion, and spatial spread of an emerging clone of methicillin-resistant *Staphylococcus aureus*. *PLoS Pathog* 2010; **6**: e1000855.

72. David S, Rusniok C, Mentasti M, Gomez-Valero L, Harris SR, Lechat P, et al. Multiple major disease-associated clones of *Legionella pneumophila* have emerged recently and independently. *Genome Res* 2016; **26**: 1555–1564.

73. Simon M, Scheuner C, Meier-Kolthoff JP, Brinkhoff T, Wagner-Döbler I, Ulbrich M, et al. Phylogenomics of *Rhodobacteraceae* reveals evolutionary adaptation to marine and non-marine habitats. *ISME J* 2017; **11**: 1483–1499.

74. Swofford DL. PAUP*. Phylogenetic Analysis Using Parsimony (*and other methods). Version 4.0b10.

75. Mitchell AL, Almeida A, Beracochea M, Boland M, Burgin J, Cochrane G, et al. MGnify: the microbiome analysis resource in 2020. *Nucleic Acids Res* 2019; **48**: D570–D578.

76. Burstein D, Amaro F, Zusman T, Lifshitz Z, Cohen O, Gilbert JA, et al. Genomic analysis of 38 *Legionella* species identifies large and diverse effector repertoires. *Nat Genet* 2016; **48**: 167–175.

77. Hemsley CM, O’Neill PA, Essex-Lopresti A, Norville IH, Atkins TP, Titball RW. Extensive genome analysis of *Coxiella burnetii* reveals limited evolution within genomic groups. *BMC Genomics* 2019; **20**: 441.

78. Challacombe JF, Petersen JM, Gallegos-Graves LV, Hodge D, Pillai S, Kuske CR. Whole-genome relationships among *Francisella* bacteria of diverse origins define new species and provide specific regions for detection. *Appl Environ Microbiol* 2017; **83**: e02589-16.

79. Meier-Kolthoff JP, Göker M. TYGS is an automated high-throughput platform for state-of-the-art genome-based taxonomy. *Nat Commun* 2019; **10**: 2182.

80. Parks DH, Chuvochina M, Rinke C, Mussig AJ, Chaumeil P-A, Hugenholtz P. GTDB: an ongoing census of bacterial and archaeal diversity through a phylogenetically consistent, rank normalized and complete genome-based taxonomy. *Nucleic Acids Res* 2022; **50**: D785–D794.

81. Richter M, Rosselló-Móra R. Shifting the genomic gold standard for the prokaryotic species definition. *Proc Natl Acad Sci U S A* 2009; **106**: 19126–19131.

82. Rao DN, Saha S, Krishnamurthy V. ATP-dependent restriction enzymes. *Prog Nucleic Acid Res Mol Biol* 2000; **64**: 1–63.

83. Oliveira PH, Touchon M, Rocha EPC. Regulation of genetic flux between bacteria by restriction-modification systems. *Proc Natl Acad Sci U S A* 2016; **113**: 5658–5663.

84. Ortiz-Severín J, Travisany D, Maass A, P. Chávez F, Cambiazo V. *Piscirickettsia salmonis* cryptic plasmids: source of mobile DNA and virulence factors. *Pathogens* 2019; **8**: 269.

85. Harms A, Brodersen DE, Mitarai N, Gerdes K. Toxins, targets, and triggers: an overview of toxin-antitoxin biology. *Mol Cell* 2018; **70**: 768–784.

86. Saavedra J, Grandón M, Villalobos-González J, Bohle H, Bustos P, Mancilla M. Isolation, functional characterization and transmissibility of p3PS10, a multidrug resistance plasmid of the fish pathogen *Piscirickettsia salmonis*. *Front Microbiol* 2018; **9**: 923.

87. Miranda CD, Zemelman R. Bacterial resistance to oxytetracycline in Chilean salmon farming. *Aquaculture* 2002; **212**: 31–47.

88. Cabello FC, Godfrey HP, Tomova A, Ivanova L, Dölz H, Millanao A, et al. Antimicrobial use in aquaculture re-examined: its relevance to antimicrobial resistance and to animal and human health. *Environ Microbiol* 2013; **15**: 1917–1942.

89. Sandoval R, Oliver C, Valdivia S, Valenzuela K, Haro RE, Sánchez P, et al. Resistance-nodulation-division efflux pump *acrAB* is modulated by florfenicol and contributes to drug resistance in the fish pathogen *Piscirickettsia salmonis*. *FEMS Microbiol Lett* 2016; **363**: fnw102.

90. Cerveau N, Leclercq S, Leroy E, Bouchon D, Cordaux R. Short- and long-term evolutionary dynamics of bacterial insertion sequences: insights from *Wolbachia* endosymbionts. *Genome Biol Evol* 2011; **3**: 1175–1186.

91. Harmer CJ, Hall RM. An analysis of the IS6/IS26 family of insertion sequences: is it a single family? *Microb Genom* 2019; **5**: e000291.

92. Naas T, Blot M, Fitch WM, Arber W. Insertion sequence-related genetic variation in resting *Escherichia coli* K-12. *Genetics* 1994; **136**: 721–730.

93. Moran NA, Plague GR. Genomic changes following host restriction in bacteria. *Curr Opin Genet Dev* 2004; **14**: 627–633.

94. Wu Y, Aandahl RZ, Tanaka MM. Dynamics of bacterial insertion sequences: can transposition bursts help the elements persist? Theories and models. *BMC Evol Biol* 2015; **15**: 288.

95. Beuzón CR, Chessa D, Casadesús J. IS200: An old and still bacterial transposon. *International Microbiology* 2004; **7**: 3–12.

96. Liu Y, Harrison PM, Kunin V, Gerstein M. Comprehensive analysis of pseudogenes in prokaryotes: widespread gene decay and failure of putative horizontally transferred genes. *Genome Biol* 2004; **5**: R64.

97. Cole ST, Eiglmeier K, Parkhill J, James KD, Thomson NR, Wheeler PR, et al. Massive gene decay in the leprosy bacillus. *Nature* 2001; **409**: 1007–1011.

98. Vigil-Stenman T, Larsson J, Nylander JAA, Bergman B. Local hopping mobile DNA implicated in pseudogene formation and reductive evolution in an obligate cyanobacteria-plant symbiosis. *BMC Genomics* 2015; **16**: 193.

99. Senchenkova SN, Popova A v., Shashkov AS, Shneider MM, Mei Z, Arbatsky NP, et al. Structure of a new pseudaminic acid-containing capsular polysaccharide of *Acinetobacter baumannii* LUH5550 having the KL42 capsule biosynthesis locus. *Carbohydr Res* 2015; **407**: 154–157.

100. Schoenhofen IC, Lunin V v., Julien J-P, Li Y, Ajamian E, Matte A, et al. Structural and functional characterization of PseC, an aminotransferase involved in the biosynthesis of pseudaminic acid, an essential flagellar modification in *Helicobacter pylori*. *Journal of Biological Chemistry* 2006; **281**: 8907–8916.

101. Hitchen P, Brzostek J, Panico M, Butler JA, Morris HR, Dell A, et al. Modification of the *Campylobacter jejuni* flagellin glycan by the product of the Cj1295 homopolymeric-tract-containing gene. *Microbiology (N Y)* 2010; **156**: 1953–1962.

102. Vinogradov E, Frimmelova M, Toman R. Chemical structure of the carbohydrate backbone of the lipopolysaccharide from *Piscirickettsia salmonis*. *Carbohydr Res* 2013; **378**: 108–113.

103. Mancilla M, Saavedra J, Grandón M, Tapia E, Navas E, Grothusen H, et al. The mutagenesis of a type IV secretion system locus of *Piscirickettsia salmonis* leads to the attenuation of the pathogen in Atlantic salmon, *Salmo salar*. *J Fish Dis* 2018; **41**: 625–634.
