# Supplementary material for: Ongoing diversification of the global fish pathogen Piscirickettsia salmonis through genetic isolation and transposition bursts
Source: ISME J. 2023 Oct 18;17(12):2247–58. doi: 10.1038/s41396-023-01531-9 (PMC10689435; doi:10.1038/s41396-023-01531-9)
Supplement: Supplementary file 2 — Supplementary Figures [file 41396_2023_1531_MOESM2_ESM.pdf]

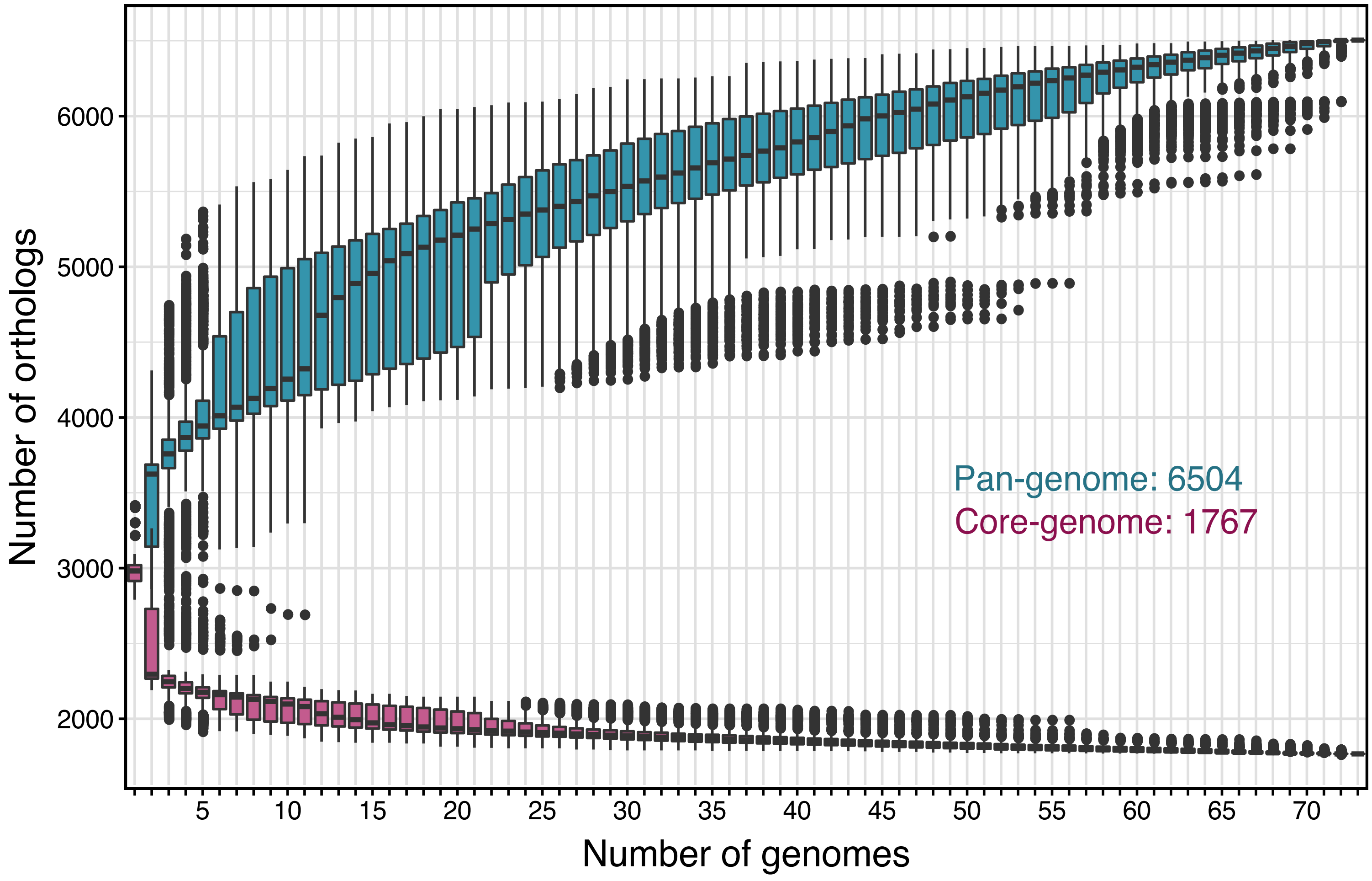

Suppl. Fig. S1. Accumulation curves for the core genome (red boxes) and pan genome (blue boxes) of the 73 *Piscirickettsia* strains. For each number of strains, the variance in homolog counts was estimated based on 1000 random selections of the given number of strains out of the entire dataset. Homolog groups of protein sequences were determined by Proteinortho. Boxes give 25 to 75% quartiles, bars in boxes the median, and whiskers 95%. Outliers are due to the large differences in gene content between the three *Piscirickettsia* genogroups.

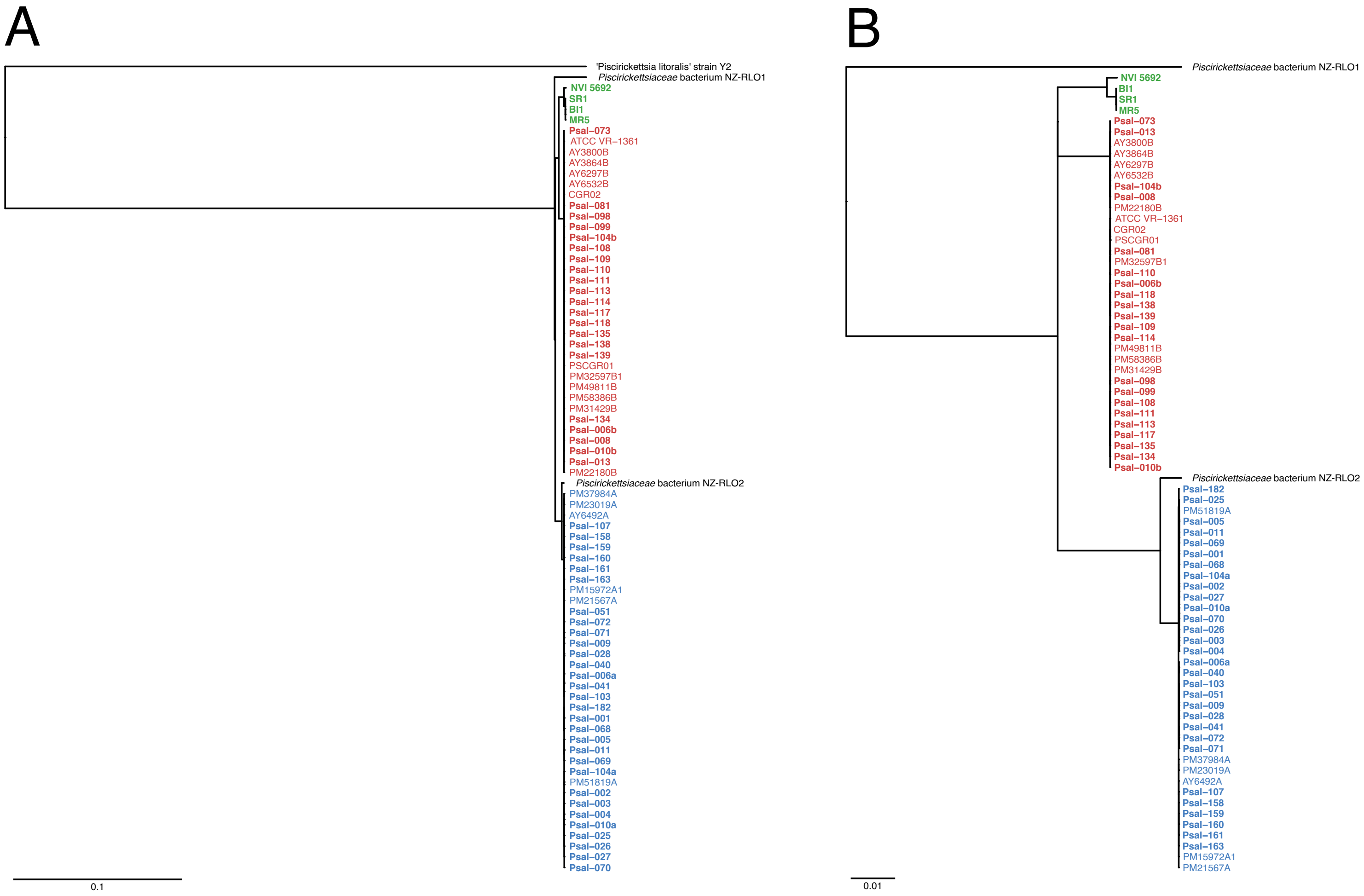

Suppl. Fig. S2. (A) Parsnp phylogenomic tree based on the core genome of all 73 *Piscirickettsia salmonis* isolates plus the three draft genome sequences that had sufficient genome coverage and which originated from New Zealand (*Piscirickettsiaceae* bacterium NZ-RLO1, NZ-RLO2) and from Hawaii (`*Piscirickettsia litoralis*´ strain Y2) (see Suppl. Table S1). Chilean isolates of the LF genogroup are depicted in red, those of the EM genogroup in blue, and Norwegian and Canadian isolates in green. Isolates printed in bold were sequenced de novo in the present study. **(B)** Parsnp phylogenomic tree without strain Y2 to show branching patterns within established genogroups. Scale bars give substitutions per nucleotide site.

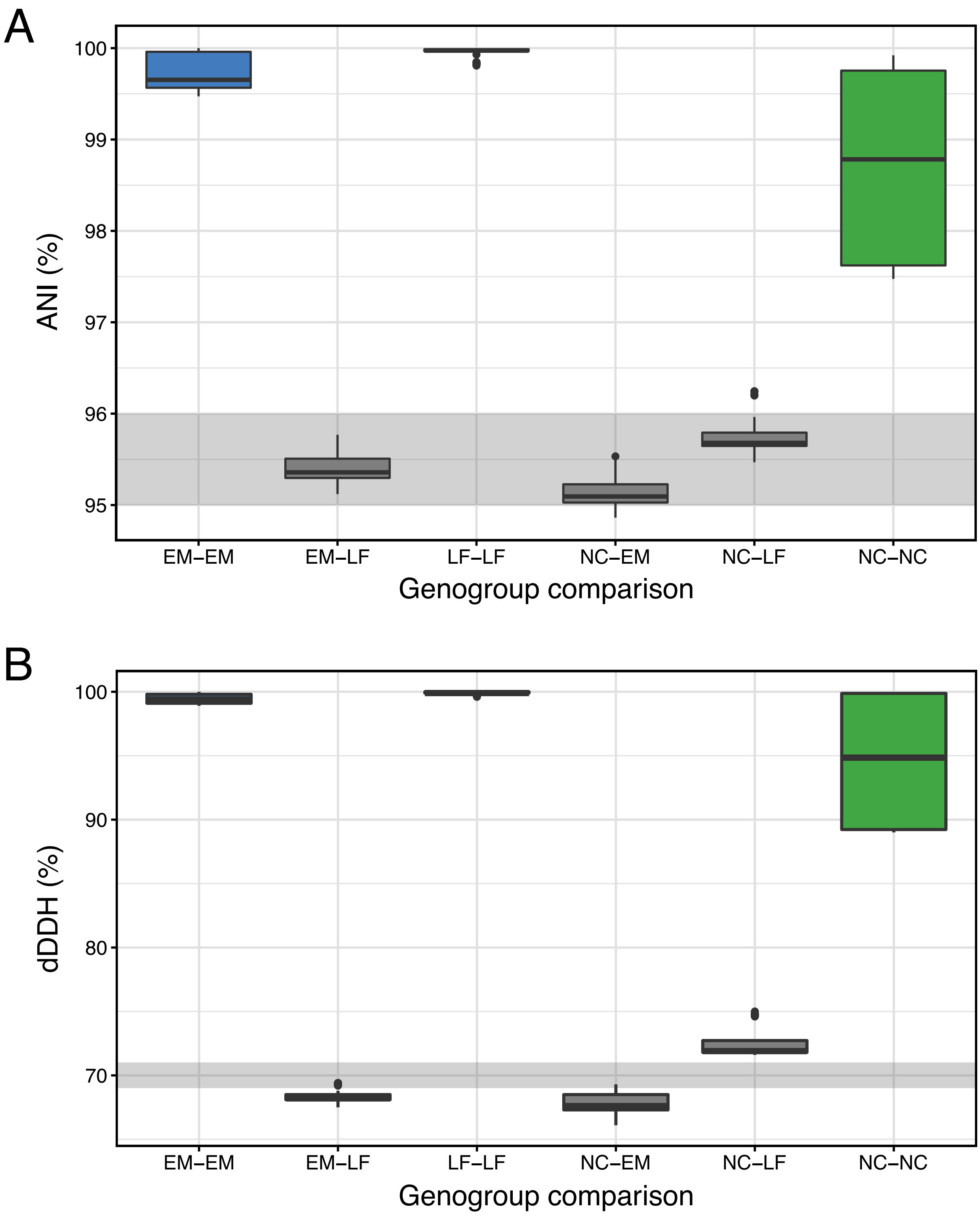

Suppl. Fig. S3. (A) Average Nucleotide Identities (ANI) of genomes calculated for all possible pairwise combinations of members from the three different *Piscirickettsia* genogroups (73 complete genomes). Grey shaded area indicates the limits for the delineation of species (95-96%). **(B)** Digital DNA-DNA hybridization values calculated for all possible pairwise combinations of members from the three genogroups. Grey shaded area indicates the limits for the delineation of species (67-73%). In both panels, bars in boxes depict the median. Lower and upper hinges of boxes correspond to the first and third quartiles (the 25th and 75th percentiles). Upper and lower whisker indicate 1.5 times the inter-quartile range (IQR, distance between the first and third quartiles). Data beyond the range of the whiskers are outliers and plotted individually.

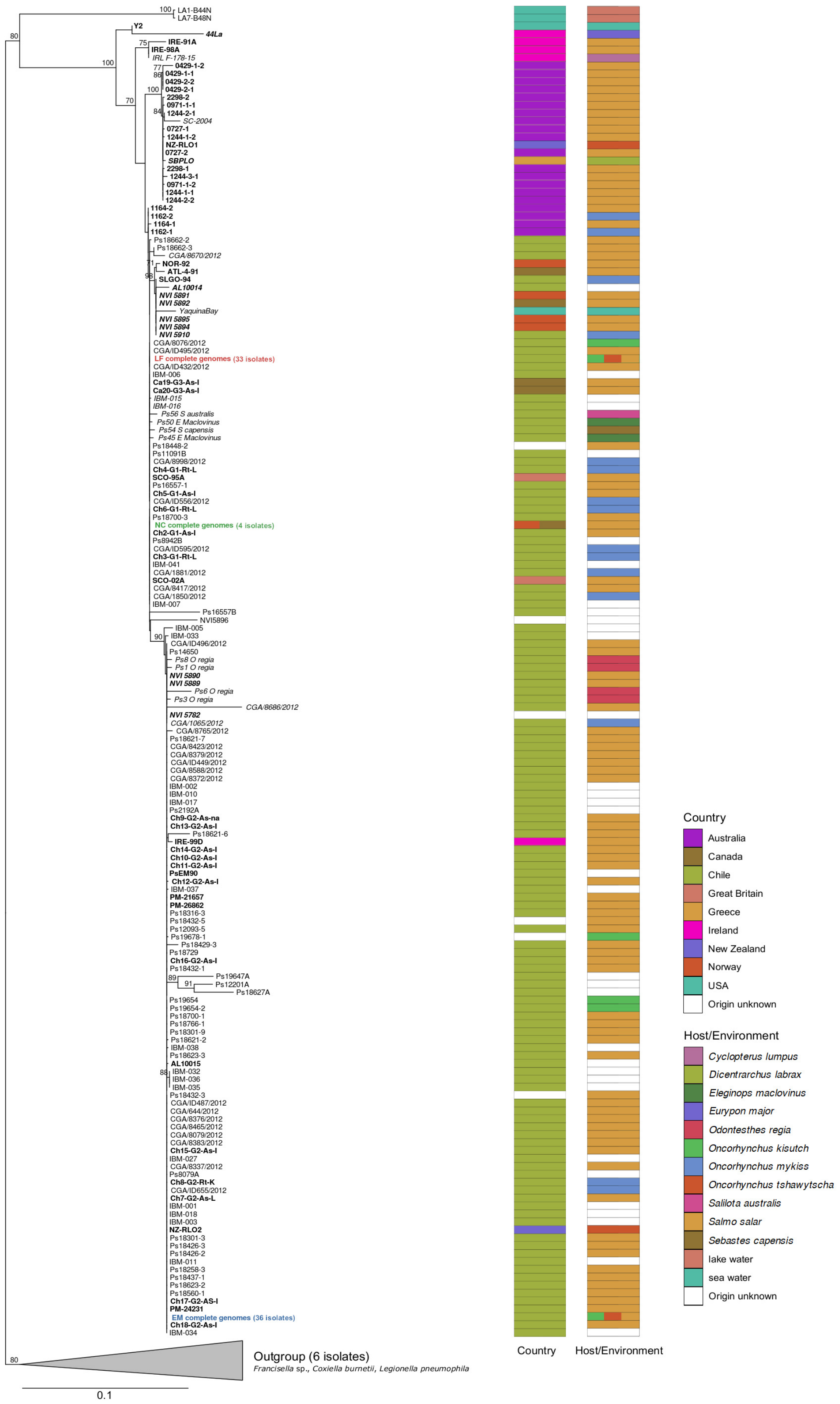

Suppl. Fig. S4. Maximum likelihood phylogenenetic tree inferred from all available 16S rRNA sequences deposited in GenBank as *Piscirickettsia salmonis*, *Piscirickettsia* sp. or *Rickettsia*-like organisms. After construction of the tree, sequences shorter than 1300 bp (indicated in italics) were inserted by maximum parsimony without changing the overall topology (see Materials and Methods). Sequences originating from cultivated bacterial strains are indicated in bold face. Sequences from 55 strains included in the comparative genomic analysis of this study are indicated in red (LF genogroup), green (NC genogoup) and blue (EM genogroup). Numbers at nodes give bootstrap values in %; only values greater than 70% are shown. Columns on the right indicate the country of origin and the host or environment of each strain, displayed by color codes. In addition to 4 salmonids (*Salmo salar*, *Oncorhynchus kisutch*, *O. mykiss*, *O. tshawytscha*) and *Dicentrarchus labrax* (European bass) in aquaculture, *Piscirickettsia* sequences were reported from wild marine fish species *Cyclopterus lumpus* (lumpfish), *Eleginops maclovinus* (Patagonian blenny), *Odonthestes regia* (Chilean silverside, pejerrey), *Salilota australis* (Patagonian cod, tadpole codling), *Sebastes capensis* (cape redfish), as well as a marine sponge (*Eurypon major*). Sequences marked as ‘Origin unknown’ lack the respective information in their GenBank entries and are not linked to a publication containing origin information.

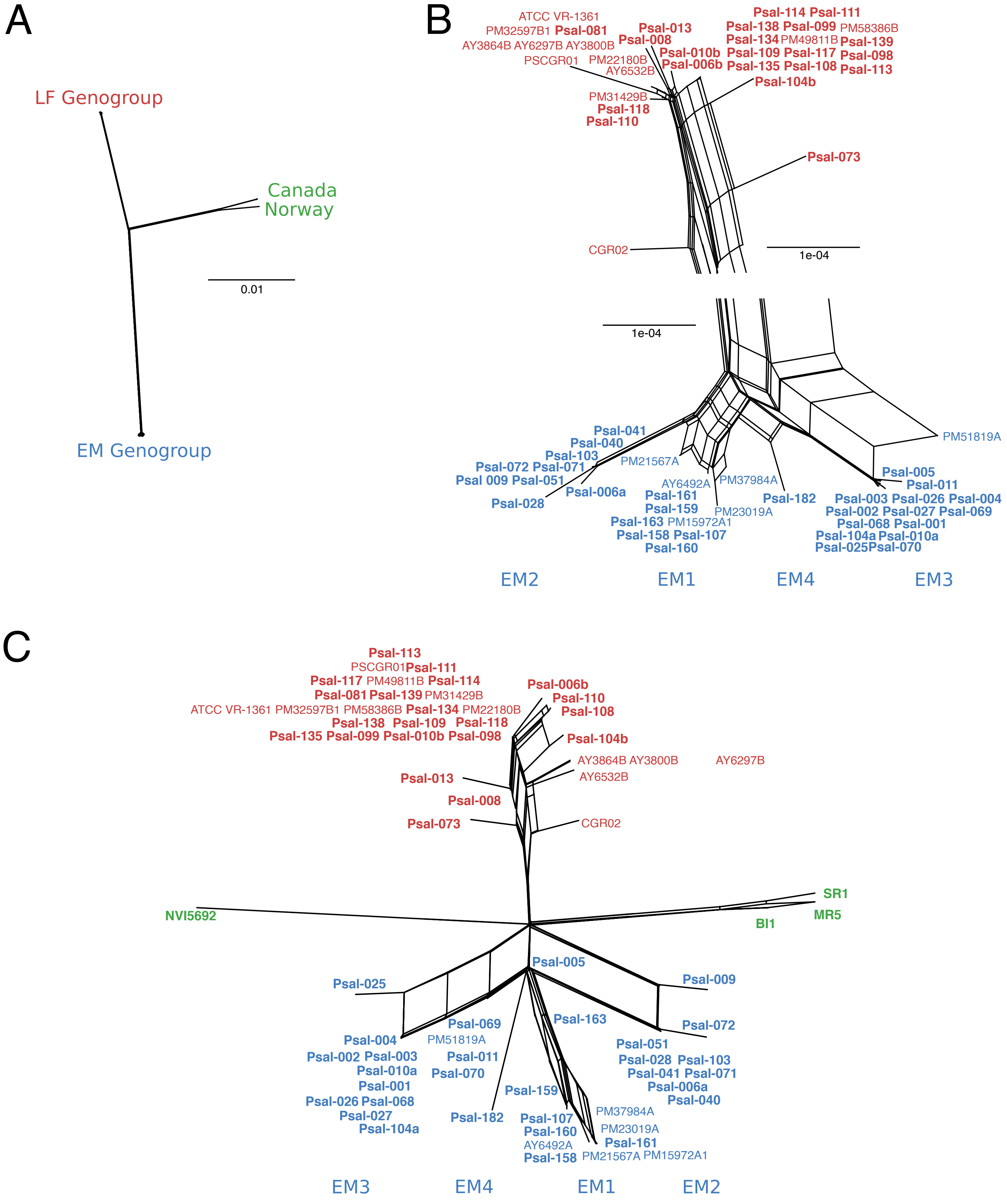

Suppl. Fig. S5. (A) Phylogenetic network inferred by SplitsTree NeighborNet analysis from the core genome alignment of the 73 *Piscirickettsia* isolates. Chilean isolates of the LF genogroup are depicted in red, those of the EM genogroup in blue, and Norwegian and Canadian isolates in green. **(B)** Details showing high resolution subnetworks of the LF genogroup and EM genogroup. **(C)** NeighborNet analysis of the presence/absence of representatives of ECE clusters in 73 *Piscirickettsia* strains. Scale bars in panels **(A)** and **(B)** give substitutions per nucleotide site**.**

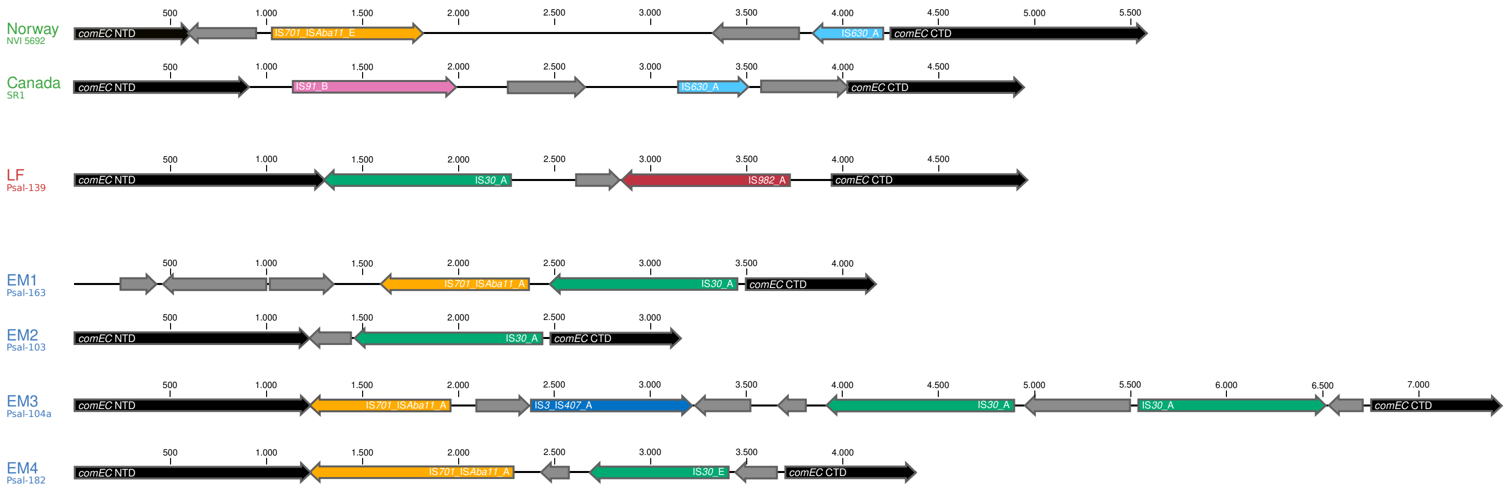

Suppl. Fig. S6. Different insertion sites and IS types in the *comEC* locus of representatives of the different *Piscirickettsia* genogroups and EM subgroups. N-terminal domains (NTD) and C-terminal domains (CTD) of *comEC* are depicted as black arrows and different IS families by arrows in different colors. Grey arrows indicate other ORFs; some of them may represent truncated transposases.

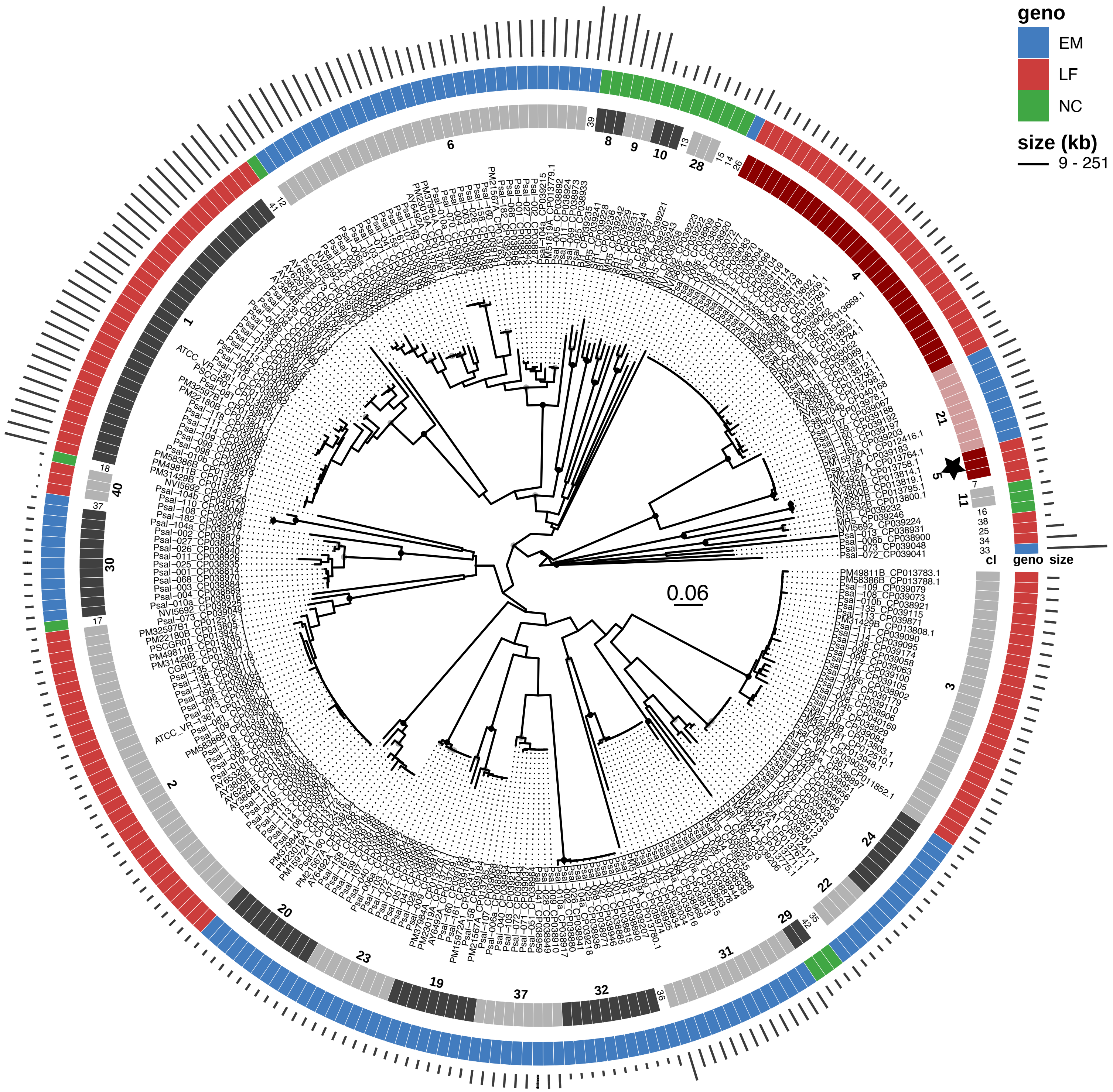

Suppl. Fig. S7. Phylogenetic tree of 290 extrachromosomal elements (ECEs) from *Piscirickettsia*. The phylogeny was calculated based on amino acid sequences with VICTOR (formula D6). Support values of 100% and between 100 and 60% are indicated by black and grey dots, respectively. ECE clusters are indicated in the inner ring (*cl*) with an alternating dark and light grey hue and the cluster number. Clusters labelled in red denote mobile ECEs as inferred from the presence of a T4SS or a relaxase. Singletons are denoted in white. The genogroup of the strains is indicated in the middle ring (*geno*) and the size of the elements in the outer ring (9 - 251 kb). The cluster of the plasmids carrying antibiotic resistance genes is indicated by a star.

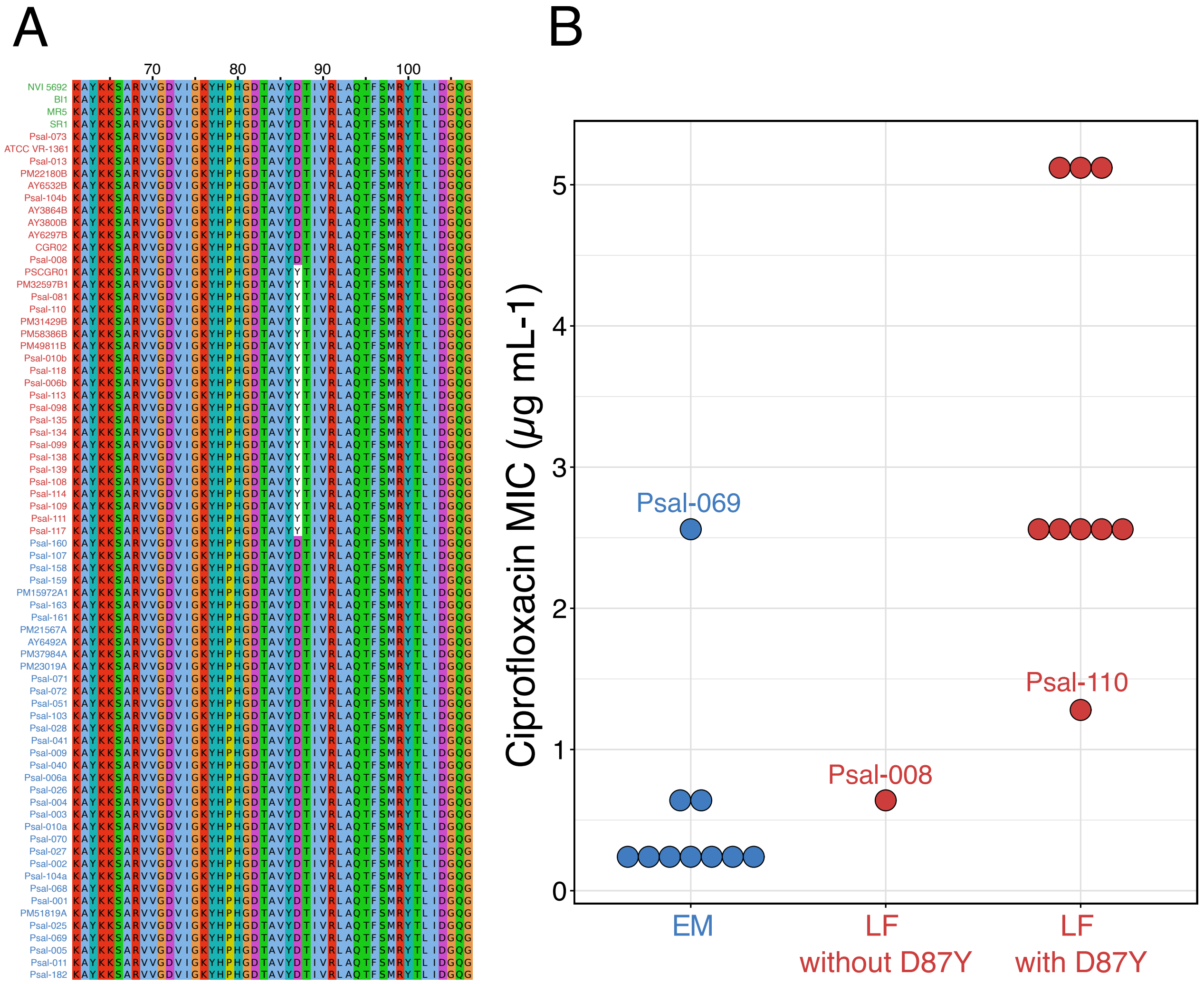

Suppl. Fig. S8. (A) Alignment of the quinolone resistance-determining region (QRDR) of the *gyrA* gene in the genomes of all 73 *Piscirickettsia* strains. The D87Y amino acid exchange is indicated. **(B)** MIC values observed for 10 strains of the EM genogroup (all lacking the *gyrA* point mutation, Psal-069 displayed a ciprofloxacin-resistant phenotype), and ten strains of the LF genogroup (Psal-008 lacking the *gyrA* point mutation). Psal-069 also did not exhibit any other known resistance mechanisms: mutations in *gyrB*, in *parC* and *parE* encoding topoisomerase IV, presence of gyrase-protecting Qnr-proteins, acetyltransferases, or high expression through mutations in promoter regions of efflux pumps [1].

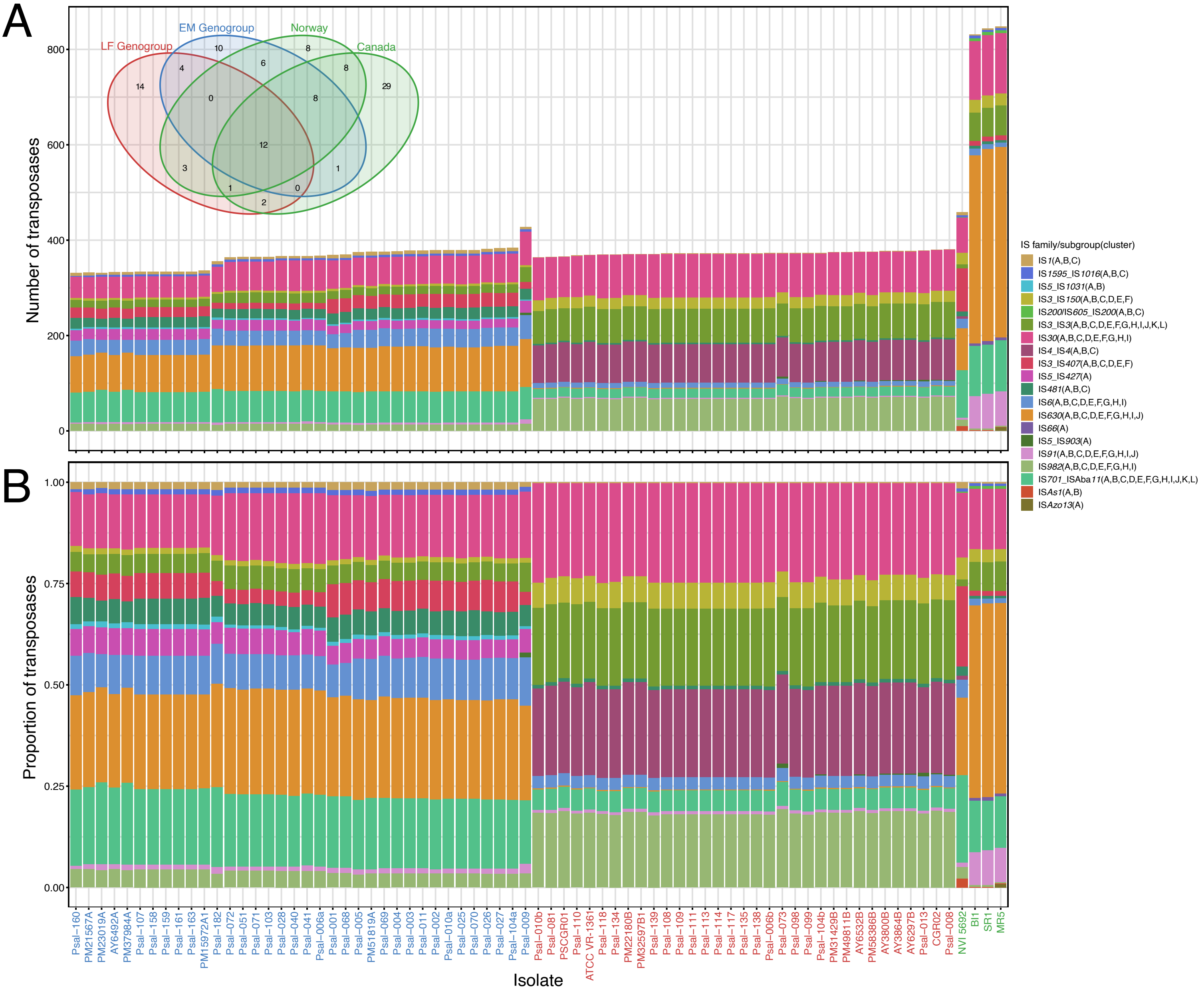

Suppl. Fig. S9. Numbers and types (IS families/subgroups, shown in different colors) of *Piscirickettsia* coding sequences identified as transposases. Transposase sequences were identified and sorted into the established IS families and subgroups by BLAST comparison against sequences in the ISfinder database [2]. **(A)** Numbers of different transposase types per *Piscirickettsia* chromosome. The Venn diagram illustrates the distribution of the 106 identified sequence clusters across the *Piscirickettsia* genogroups. Up to 12 different sequence clusters (denoted A-L in the legend) could be distinguished in individual IS families/subgroups. **(B)** Proportions of different transposase types in each *Piscirickettsia* chromosome. See Suppl. Table S8 for further details on the IS elements.

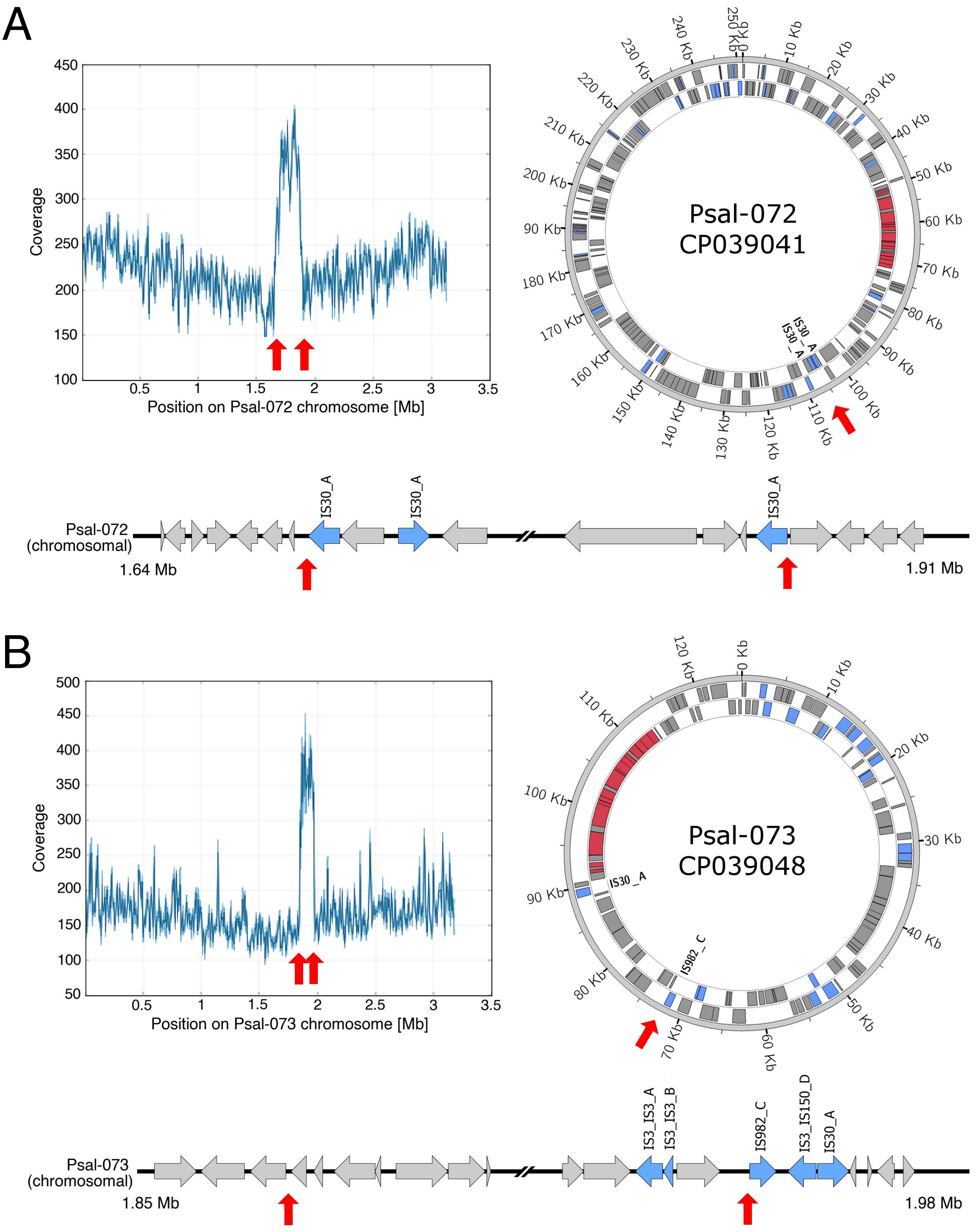

Suppl. Fig. S10. Observed coverage of PacBio sequencing reads along the chromosomes, circular representation of ECEs carrying *icm/dot* gene clusters and visualization of the chromosomal excision sites. **(A)** EM genogroup strain Psal-072. **(B)** LF genogroup strain Psal-073. In both genomes, the region surrounding one of the *icm/dot* gene clusters (260 kb in Psal-072 and 125 kb in Psal-073) showed twofold coverage and could be assembled into circular extrachromosomal elements (ECE-type 33/ Psal-072_CP039041 and ECE-type 34/Psal-073_CP039048; compare Suppl. Table S4). These composite transposons comprise *icm/dot* genes (colored in red) and various ISs (colored in blue). Individual icm/dot genes are listed in Suppl. Table S4B and S4C. Red arrows indicate excision sites on the chromosomes which are located at IS elements (two IS*30*_A in Psal-072 and one IS*982* in Psal-073, respectively).

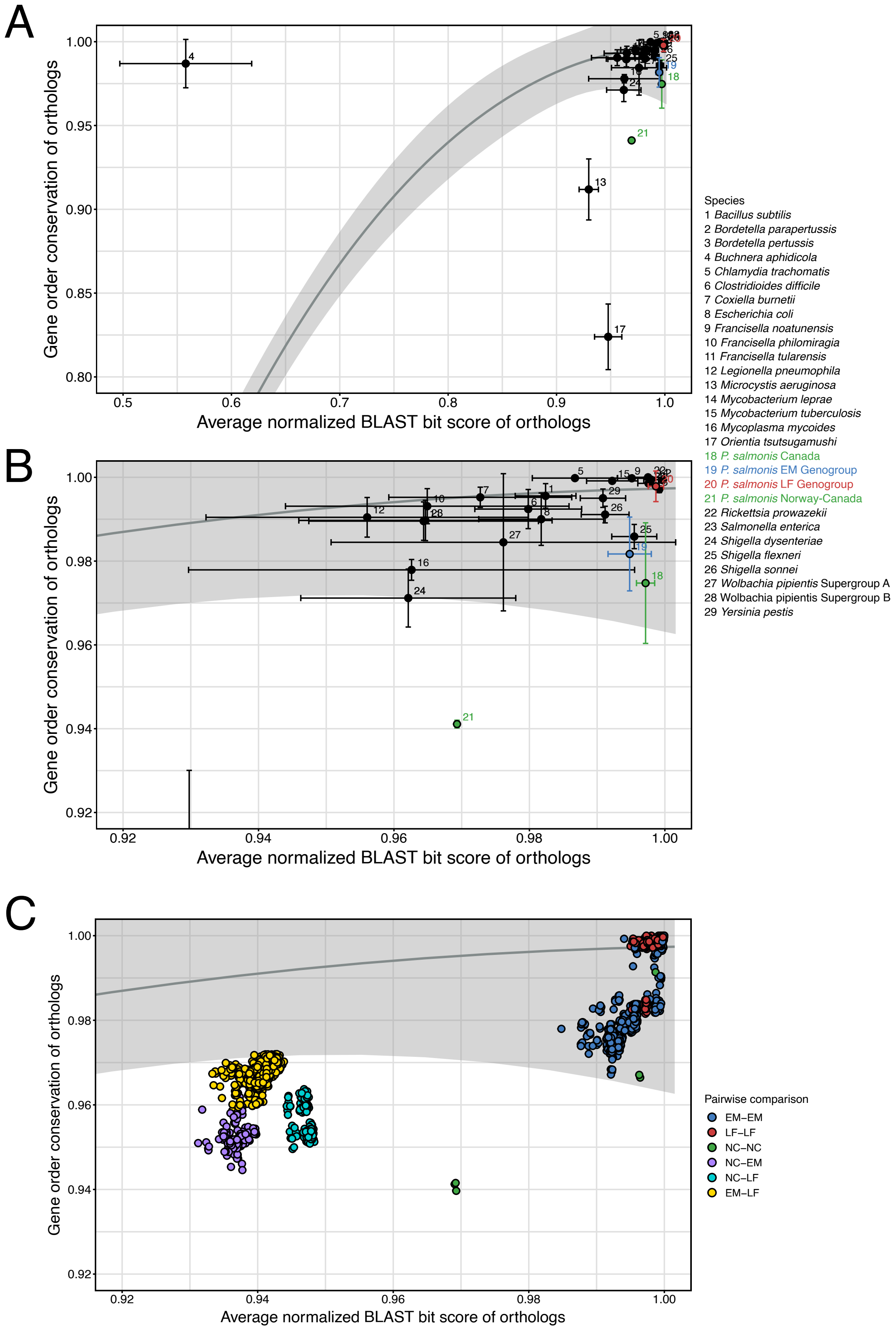

Suppl. Fig. S11. Gene order conservation (GOC) of orthologs plotted against average sequence similarity (average normalized BLAST bit score). Calculations were made for all 73 *Piscirickettsia* chromosomes and four chromosomes respectively for selected reference species including species with high transposase content (compare Fig. 2). For comparison, a regression (grey curve) and the 95% confidence interval (area shaded in grey) for data points previously published for pairwise comparisons between 634 archaeal and bacterial genomes [3] is shown. **(A)** Overview over within-species/within-genogroup comparisons for *Piscirickettsia* and all reference species. For each species, the mean of the GOC is plotted against the mean of the BLAST bit scores and for each variable one standard deviation is depicted. **(B)** Detailed view of species with high GOC and high similarity values. **(C)** Individual data plotted for all pairwise comparisons of the 73 *P. salmonis* chromosomes. Data points for *P. salmonis* are colored by genogroup affiliation.

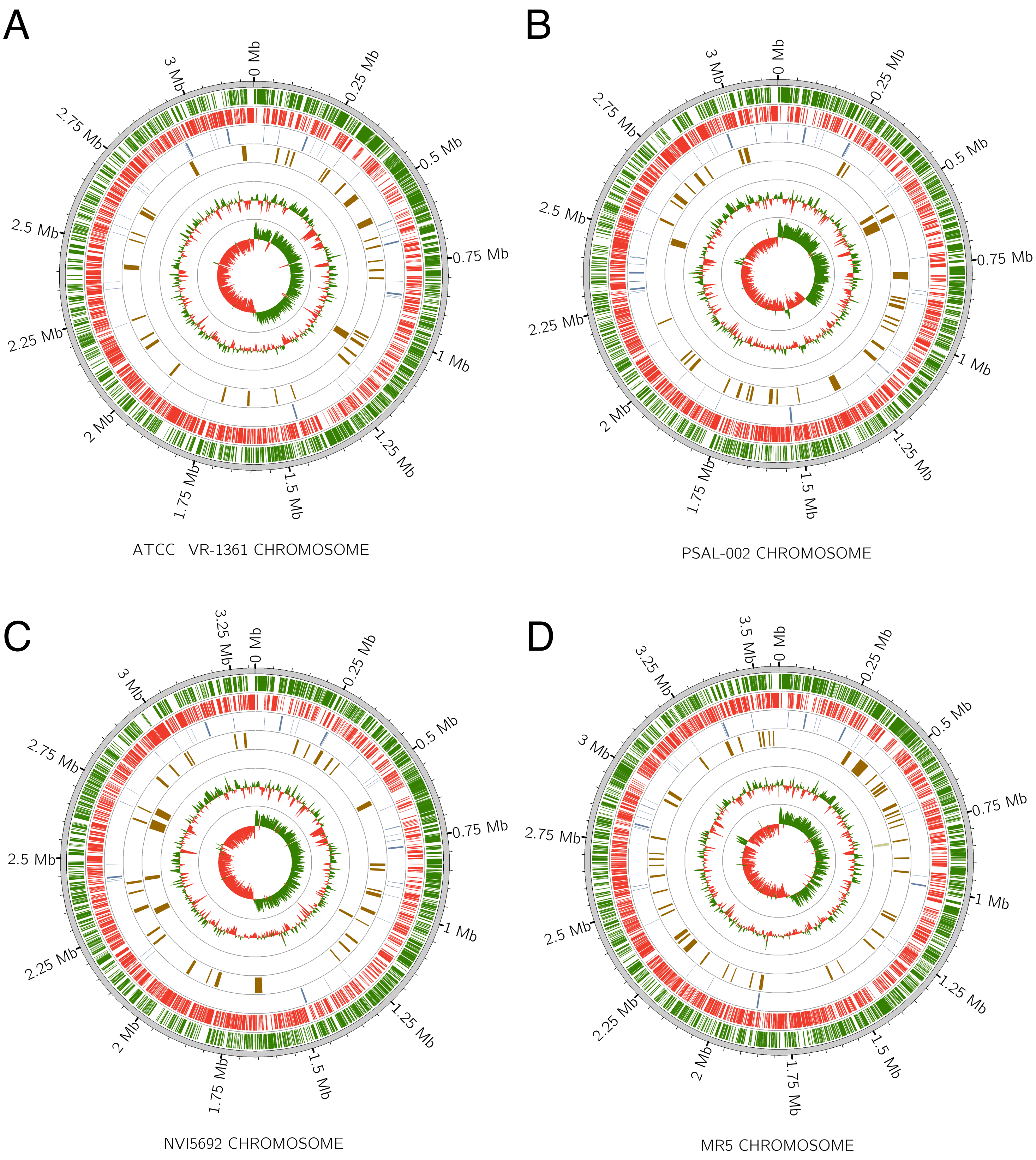

Suppl. Fig. S12. Circular illustrations of representative chromosomes of different *Piscirickettsia* genogroups. Circos plots show (from outer to inner circles) CDS on plus strand (green), CDS on minus strand (red), RNA (blue), genomic islands (brown), phages (light brown), GC content (green, >0; red, <0), and GC skew (green, >0; red, <0). **(A)** ATCC VR-1361 (LF genogroup). **(B)** Psal-002 (EM genogroup). **(C)** NVI5692 (Norway). **(D)** MR5 (Canada).

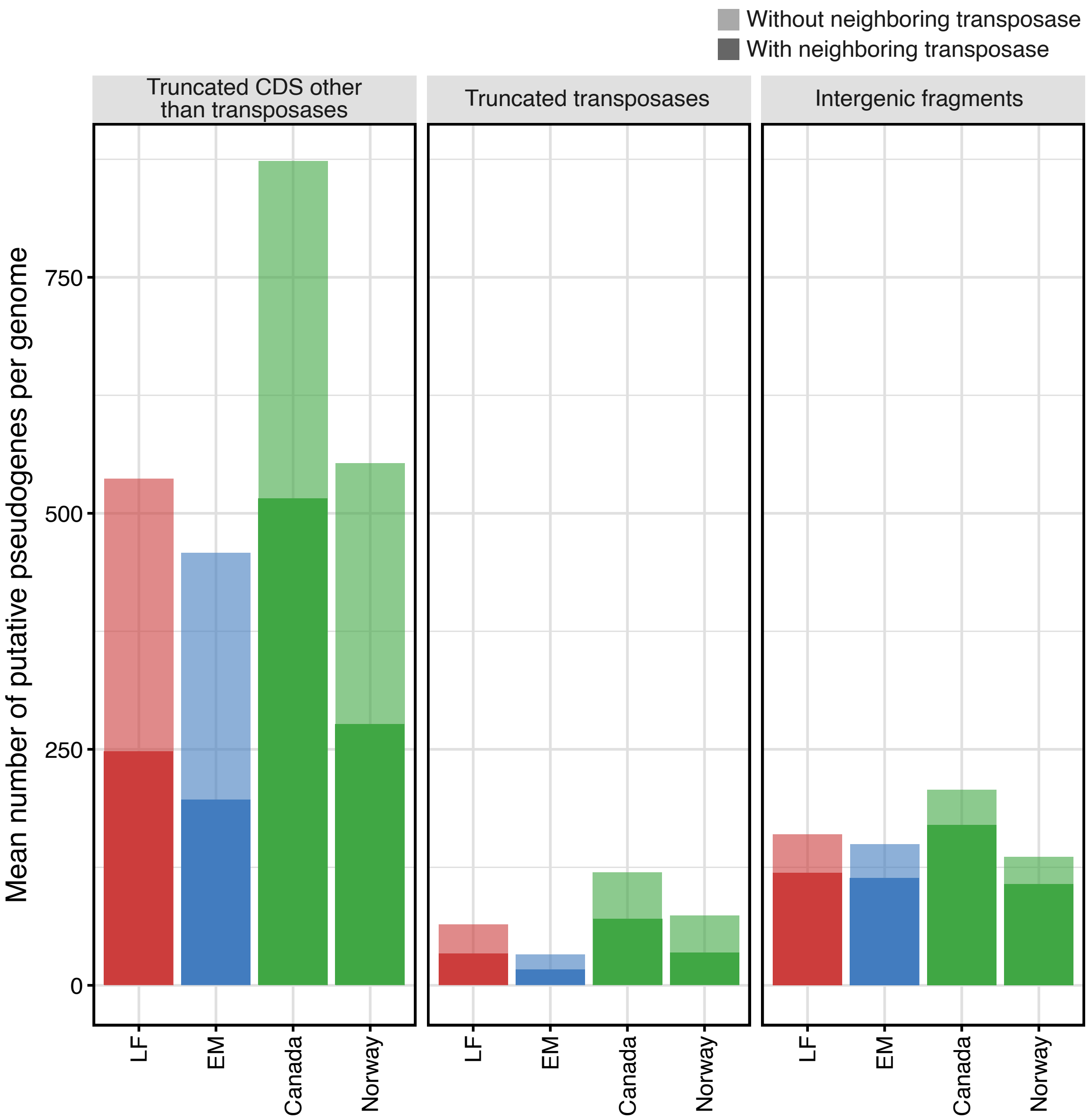

Suppl. Fig. S13. Mean numbers of the three different types of putative pseudogenes per genome identified in the different *Piscirickettsia* genogroups. Darker and lighter colored parts of columns give numbers of pseudogenes with and without an adjacent transposase gene, respectively.

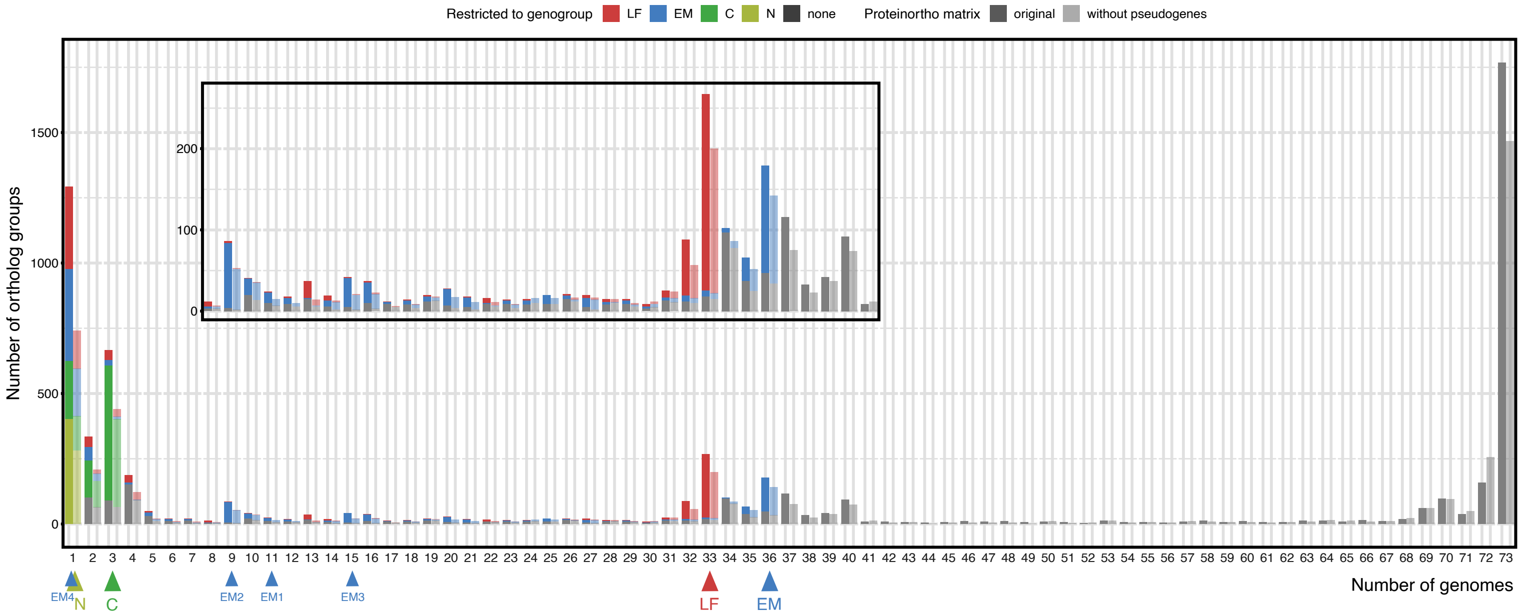

Suppl Fig. S14. Distribution of the *Piscirickettsia* pan genome among the 73 strains. Depicted is the frequency with which groups of homologs occur exclusively in a given number of genomes. For each number of genomes, frequencies are shown for all homologs (left bars) and for the homologs without pseudogenes (right bars). Numbers of homolog groups that are restricted to an individual geno- or subgroup are shown in color. The highest numbers of homologs are shared by all genomes and represent the core genome. The second highest number of homologs occur in only a single genome. Additional peaks in the frequency are observed at numbers of genomes that correspond to the sizes of the different geno- or subgroups (3 Canadian genomes, 33 genomes of the LF genogroup, 36 genomes of the EM genogroup) and the sizes of EM-subgroups (EM1, 11 genomes; EM2, 9 genomes; EM3, 15 genomes) (indicated by colored arrows). Insert, enlarged view of the section between 8 and 41 genomes.

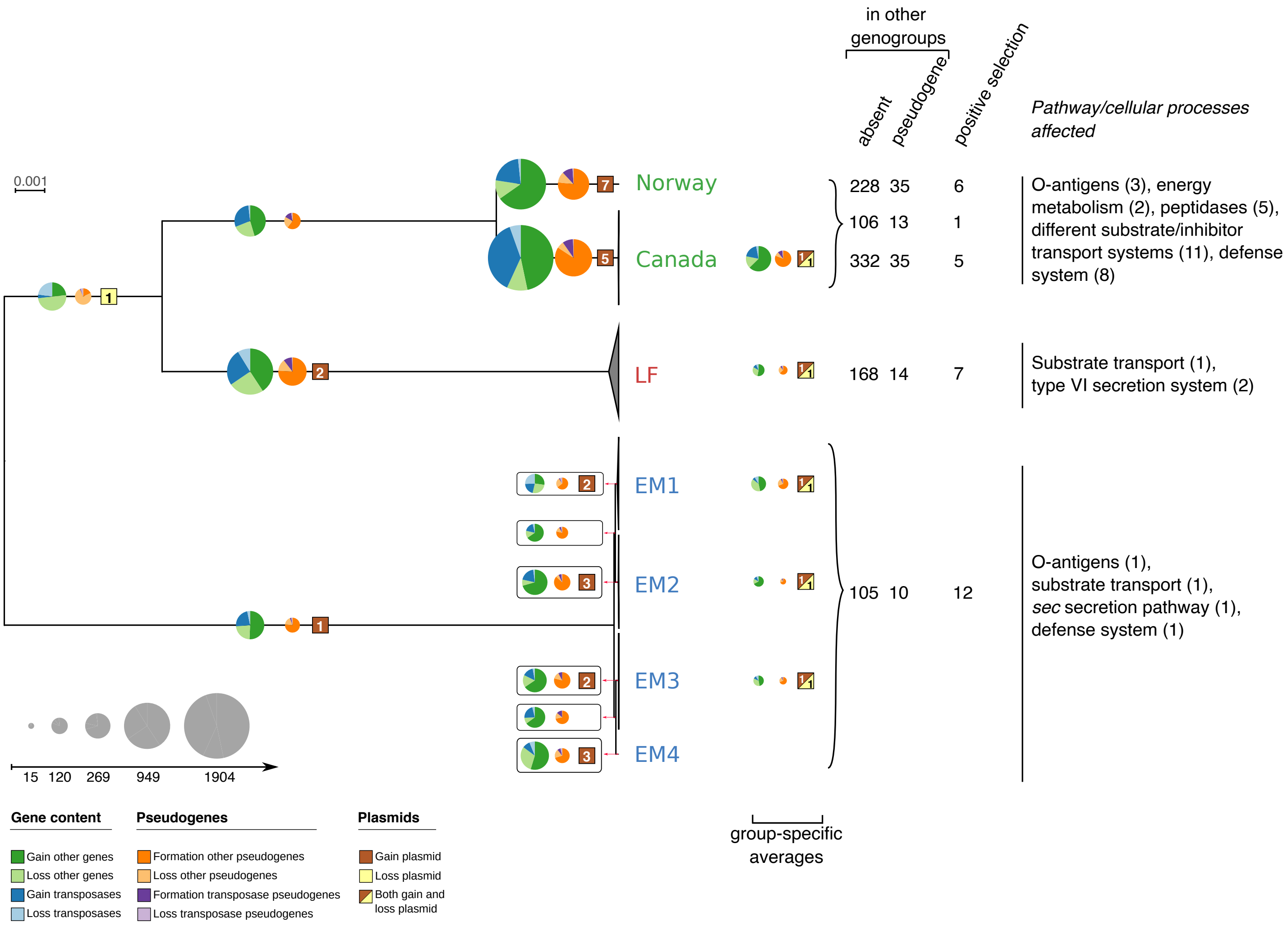

Suppl. Fig. S15. Changes in gene content during the evolution of the Piscirickettsia geno- and subgroups. Ultrametric phylogenomic tree with a quantitative comparison of changes in gene content (pie charts with blue and green colors for transposases and other genes, respectively), formation and loss of transposase and other pseudogenes (pie charts with purple and orange colors), and of changes in extrachromosomal elements (ECEs; squares shaded with brown and yellow color) during the different phases (i.e. subbranches) of the speciation of the genus Piscirickettsia. For terminal radiations in the individual genogroups or EM subgroups, average values for gains and losses are provided next to geno-/subgroup designations. The relative area of pie charts is proportional to the square root of the number of gene/plasmid gains and losses. The scaling of pie charts with mean numbers are provided at the bottom. Missing squares indicate that no changes occurred on the respective branches. Numbers of genes that are group-specific due to their absence or pseudogenization in other genomes or showing signs of positive selection are given for the three genogroups and, separately, for the Norwegian and Canadian strains. Also, major pathways and cellular processes inferred from the annotated genes as affected by the absence, pseudogenization or positive selection are provided for the three genogroups (see text for further details).

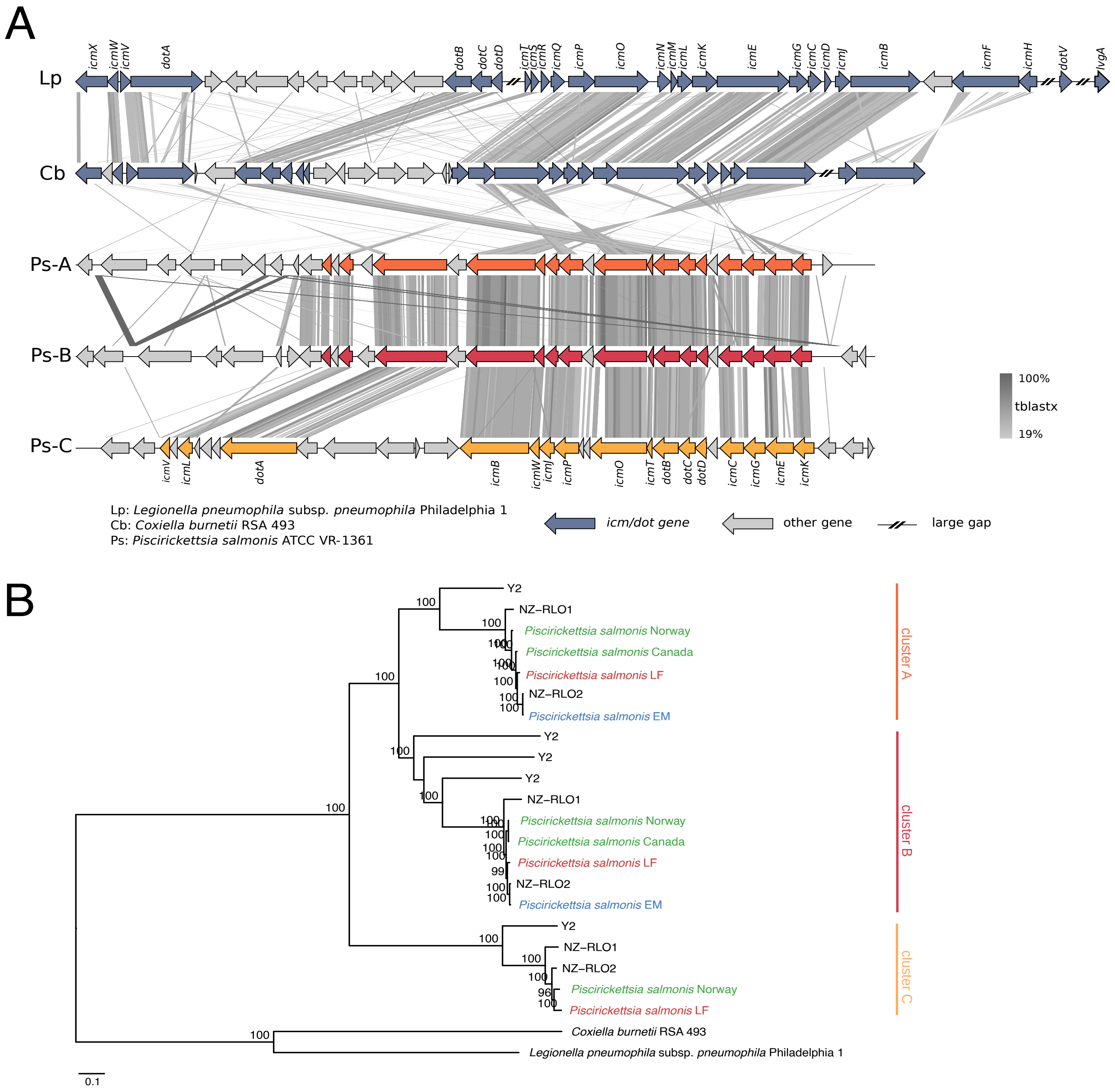

Suppl. Fig. S16. (A) Structure and BLAST identity of the *icm/dot* gene clusters in different *Piscirickettsia* genogroups as compared to the clusters in *L. pneumophila* subsp. *pneumophila* strain Philadelphia 1^{T} (GenBank: AE017354.1) and *C. burnetii* strain RSA 493 (GenBank: AE016828.3). **(B)** Phylogenetic tree as inferred from concatenated alignments of the core *icm/dot* genes *dotA*, *dotB*, *dotC*, *dotD*, *icmB*, *icmC*, *icmE*, *icmG*, *icmJ*, *icmK*, *icmO*, *icmP*, *icmT*, *icmV* and *icmW*, including *icm/dot* clusters found in the draft genomes of the deep branching lineages NZ-RLO1, NZ-RLO1 and Y2. The most deeply branching genome of *Piscirickettsia* strain Y2 harbors homologous regions to all three *icm/dot* clusters (A – MDTU01000001.1 1135883-1168807; C – MDTU01000001.1 1105575-11355882). Cluster B is present on three different scaffolds, possibly due to assembly errors.

1. Sandoval R, Oliver C, Valdivia S, Valenzuela K, Haro RE, Sánchez P, et al. Resistance-nodulation-division efflux pump *acrAB* is modulated by florfenicol and contributes to drug resistance in the fish pathogen *Piscirickettsia salmonis*. *FEMS Microbiol Lett* 2016; **363**: fnw102.

2. Siguier P. ISfinder: the reference centre for bacterial insertion sequences. *Nucleic Acids Res* 2006; **34**: D32–D36.

3. Yelton AP, Thomas BC, Simmons SL, Wilmes P, Zemla A, Thelen MP, et al. A semi-quantitative, synteny-based method to improve functional predictions for hypothetical and poorly annotated bacterial and archaeal genes. *PLoS Comput Biol* 2011; **7**: e1002230.
